# Cluster‐Mediated Solute Stabilization and Shear‐Bypass Synergistic Strengthening in High‐Alloyed Systems

**DOI:** 10.1002/advs.202522466

**Published:** 2026-03-06

**Authors:** Wei Yu, Yu Bai, Jiawen Li, Kaixi Jiang, Hai Hao, Tao Jiang, Shunan Zhang, Yong Li, Zhaodong Wang, Kai Zhao, Shiwei Li

**Affiliations:** ^1^ Key Laboratory of Solidification Control and Digital Preparation Technology (Liaoning Province) School of Materials Science and Engineering Dalian University of Technology Dalian China; ^2^ Shandong Laboratory of Aluminum Advanced Manufacturing in Binzhou Weiqiao‐UCAS Science and Technology Park Binzhou China; ^3^ Ningbo Research Institute of Dalian University of Technology Ningbo China; ^4^ School of Materials and Metallurgy University of Science and Technology Liaoning Anshan China; ^5^ State Key Laboratory of Rolling and Automation Northeastern University Shenyang China; ^6^ Guangxi Advanced Aluminum Processing Innovation Center Co., Ltd Nanning China

**Keywords:** Al‐Li alloys, multiscale simulation, process window, shear‐bypass synergistic strengthening, solute segregation, twin‐roll casting

## Abstract

High alloy content and multiphase synergistic strengthening have become crucial strategies in contemporary metallic materials design for performance enhancement. A major challenge in this field lies in addressing compositional segregation arising from differences in solute diffusion, especially‌ in high‐alloy systems such as aluminum‐lithium (Al‐Li) alloys. This study explores the 2060 Al‐Li alloy, a representative high‐alloy system, produced using twin‐roll casting, a segregation‐sensitive process, and analyzes segregation behavior through a customized solute field model. The results reveal that nanoclusters with high solute tolerance effectively reduce segregation‐induced degradation, leading to significant improvements in both strength and ductility, with an 18.7% increase in strength and a 41.5% increase in elongation. Furthermore, a shear‐bypass synergistic strengthening mechanism was identified. This work not only unlocks the potential of the twin‐roll casting process by leveraging sub‐rapid solidification advantages but also offers a promising technical and theoretical approach to mitigating compositional segregation, addressing a long‐standing challenge within the high‐alloy metallic materials industry.

## Graphical abstract

This study demonstrates that in high‐alloy systems, nanoclusters with high solute tolerance can effectively suppress compositional segregation. This mitigation leads to a concurrent 18.7% increase in strength and 41.5% improvement in ductility, which is attributed to a shear‐bypass synergistic strengthening mechanism. The findings provide a new pathway for enhancing properties in segregation‐prone alloys.

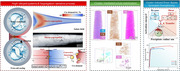

## 1 Introduction

In modern metallic materials design, achieving high alloy content coupled with multiphase synergistic strengthening has become pivotal for enhancing material properties [1, 2, 3]. By engineering synergistic interactions between various precipitate phases and solute elements, materials can achieve a balance of lightweight properties, high strength, toughness, and multifunctionality [4]. However, as the concentration of alloying elements increases, the complexity of controlling solidification behavior also escalates. In industrial production, especially under conditions of low cooling rates, the differences in the diffusion behaviors between fast‐ and slow‐diffusing solute elements become pronounced, resulting in compositional segregation and microstructural instability [5]. Fast‐diffusing solutes tend to migrate quickly to grain boundaries at elevated temperatures, forming coarse precipitates and inducing localized coarsening. In contrast, slow‐diffusing solutes are unable to effectively participate in early‐stage crystal growth and phase transformations, resulting in solute depletion in the matrix and destabilization of precipitates [6]. These challenges significantly narrow the processing window for high‐alloy materials, posing substantial obstacles to materials design and manufacturing on a global scale [7, 8]. Such challenges are particularly evident in high‐alloy Al‐Li systems, where severe compositional segregation hinders their broader application in aerospace.

To address these challenges, sub‐rapid solidification technology (cooling rate between 10^2^°C/s–10^3^°C/s) has emerged as a promising solution [9]. By accelerating cooling rates and enhancing solidification driving forces, this approach refines microstructures, broadens the processing window for high‐alloy systems, and mitigates diffusion‐driven segregation [10]. Among sub‐rapid solidification technologies, twin‐roll casting (TRC) stands out for its short process, low cost, and suitability for producing large‐scale sheets, combining both scientific and commercial value. Ideally, TRC can achieve a uniform microstructure through rapid solidification. However, in practical production, although TRC enhances solute solubility and alleviates solute diffusion conflicts, it inevitably induces complex macrosegregation phenomena such as stress‐induced segregation and solute channeling [11, 12]. In this continuous forming process, which transitions from liquid to solid, solutes enriched at the solid‐liquid interface are highly susceptible to the effects of rheological stress and solute transport dynamics, exacerbating macrosegregation and undermining material strength and ductility [13]. Therefore, the segregation‐sensitive twin‐roll casting (TRC) process serves as an effective research platform to address the industrial challenge of composition control in high‐alloy Al‐Li systems. More critically, deciphering segregation mechanisms within the TRC process and establishing robust TRC protocols for high‐alloy Al alloys not only unlocks the full potential of sub‐rapid solidification but also pioneers a transformative pathway toward cost‐effective, high‐strength‐toughness Al‐Li alloys.

Existing approaches to mitigate segregation, such as homogenization, large‐scale deformation, or nanoscale grain refinement, have inherent drawbacks [14, 15, 16]. Homogenization is often inadequate for complex high‐alloy compositions, especially under macro‐segregation conditions. Large‐scale deformation methods like accumulative roll bonding can reduce segregation but undermine TRC's short‐process advantages by adding complexity and cost. Nanoscale grain refinement improves uniformity but elevates costs and complicates process feasibility due to challenges in cluster control.

Beyond downstream processing, interventions at the solidification stage are pivotal, particularly those involving alloy modification and the application of external fields. Although parameter optimization and grain refiners enhance nucleation [17, 18], their capacity to eradicate centerline segregation in high‐solute alloys remains constrained by the severe partition coefficients of solutes. Consequently, active control via external fields, including electromagnetic stirring (EMS) and ultrasonic treatment, has been integrated into the TRC process [19, 20, 21]. These techniques introduce body forces such as Lorentz forces or acoustic streaming and cavitation into the melt pool. These physical interactions induce forced convection that homogenizes the thermal‐solute field and mechanically fractures the primary dendritic network. This dynamic perturbation promotes a columnar‐to‐equiaxed transition (CET) and disrupts the continuous solute‐rich channels, thereby significantly alleviating macro‐segregation.

In contrast, nanoscale clusters exhibit unique advantages in mitigating solute segregation by suppressing the solute diffusion conflict. Unlike homogenization or large‐scale deformation, which often fail to achieve uniformity or introduce additional complications and costs, clusters can be effectively regulated through straightforward heat treatments [22]. As demonstrated by Liu et al. [23, 24], these clusters possess a high tolerance for compositional variations, making them particularly suitable for managing heterogeneities in high‐alloy Al‐Li systems. This capability enables an effective balance between strength and ductility even within segregated microstructures. Furthermore, such a cluster‐based strategy is well‐suited for short‐process techniques like TRC, where minimizing downstream processing is crucial. By integrating cluster formation directly into the TRC workflow, targeted strengthening could be achieved with minimal added complexity.

To rigorously evaluate the efficacy of this mechanism in high‐alloyed systems, the 2060 Al‐Li alloy, characterized by a wide solidification range and high sensitivity to segregation, was selected as a representative model. In this work, the TRC process was employed for fabrication, and a customized solute field model was developed to quantitatively analyze the underlying mechanisms of segregation and the counteracting effects of cluster regulation. By regulating precipitates into clusters, needle‐like structures, and over‐coarsened states, we systematically studied their individual contributions to the strength and toughness of highly segregated microstructures. Furthermore, a detailed analysis of the shearing and bypass mechanisms involved in dislocation‐cluster interactions was conducted. This study not only addresses the degradation of material properties due to segregation under rapid cooling but also advances our understanding of the connection between microstructure evolution and macroscopic properties. By elucidating how cluster strengthening mitigates segregation, this work provides new process designs and theoretical models for nanoscale control, offering innovative solutions for the production and application of high‐alloy materials.

## 2 Material and methods

### 2.1 Sample Preparation

In this study, 2060 Al‐Li alloy was prepared using both the Twin‐Roll Casting (TRC) process and the conventional Water‐Cooled Copper Mold Casting (MC) process for comparison. The alloy composition was Al‐3.95Cu‐0.75Li‐0.85Mg‐0.4Zn‐0.3Mn‐0.25Ag‐0.11Zr (wt.%). High‐purity raw materials were used, including commercial purity Al (99.7 wt.%), Li (99.9 wt.%), Zn (99.9 wt.%), Mg (99.9 wt.%), and Ag (99.9 wt.%), as well as Al‐5Zr, Al‐10Mn, and Al‐50Cu master alloys. All materials, except for Li, were melted into the Al matrix at 720°C, followed by degassing using hexachloroethane. To minimize oxidation and explosion risks, a 30% LiF–70% LiCl cover flux was applied, and pure Li, wrapped in Al foil, was introduced into the melt. The melt temperature was stabilized at 705°C for 10 mins prior to casting. The melt was then cast into water‐cooled copper molds (200 × 400 × 5 mm) for the MC samples, while TRC sheets were produced with a cast‐rolling speed of 1.05 m/min and a roll gap height of 5 mm. All samples underwent homogenization at 470°C for 24 h. Subsequently, hot rolling was performed at 430°C. To avoid cracking caused by excessive temperature loss during multi‐pass processing, a two‐pass reduction schedule (5 mm‐4 mm‐3 mm) was adopted. Finally, the hot‐rolled sheets were cold rolled from 3 mm to 1.5 mm with a precise reduction of approximately 0.033 mm per pass. A solution treatment was performed at 490°C for 1 h, followed by water quenching and 6% pre‐stretching. Three distinct aging treatments were applied to the TRC samples to examine precipitation behavior:

First Peak‐Aged Sample (FA): 155°C for 35 h.

Second Peak‐Aged Sample (SA): 155°C for 46 h.

Over‐Aged Sample (OA): 155°C for 60 h.

### 2.2 Structural Characterization and Mechanical Testing

The microstructural characteristics of the samples were examined using multiple techniques to comprehensively understand their properties. An Electron Probe Microanalyzer (EPMA) was used to analyze the distribution of alloying elements. The texture of the samples was evaluated through Electron Backscatter Diffraction (EBSD), with EBSD samples prepared by electropolishing in a solution of 10% HClO_4_ and 90% C_2_H_5_OH. Transmission Electron Microscopy (TEM) was utilized to study precipitation behavior, with TEM samples prepared via twin‐jet polishing in a solution of 30% HNO_3_ and 70% CH_3_OH at 15 V, maintaining temperatures between −25 to −30°C. TEM analysis was conducted using an FEI G2 F20 TEM, and data from EBSD and TEM were processed using AZtecCrystal and Digital Micrograph software, respectively. The quantitative statistics of precipitate size and number density (Figures 5, 6, 7) were derived from TEM images using ImageJ software. To mitigate the stereological errors inherent in 2D projections of 3D microstructures, a strict ‘edge‐on’ observation criterion was applied. Only precipitates exhibiting sharp, needle‐like contrast (aligned parallel to the electron beam) were selected for length and thickness measurements, while inclined precipitates were excluded to avoid projection broadening effects. A minimum of 50 particles were measured for each state. It is acknowledged that while this 2D method yields projected dimensions, the consistent imaging conditions across all samples ensure that the relative trends in precipitate evolution are statistically reliable.

To further explore the deformation and fracture mechanisms during the tensile process, Digital Image Correlation (DIC) analysis was employed using a high‐speed camera and VIC‐3D DIC software. The high‐resolution strain maps provided by DIC enabled an in‐depth analysis of strain localization and damage evolution, linking mechanical behavior with microstructural features. To probe the atomic‐scale solute distribution and clustering behavior, atom probe tomography (APT) analysis was performed. Needle‐shaped specimens were fabricated site‐specifically from the FA sample using a standard lift‐out technique via a Focused Ion Beam (FIB) system. To ensure the representativeness of the matrix strengthening mechanism, the tips were extracted specifically from the intragranular regions at the mid‐thickness of the sheet, deliberately avoiding macroscopic segregation bands and coarse intermetallic compounds. The APT acquisitions were subsequently conducted on a CAMECA LEAP 6000 XR local electrode atom probe operating in laser‐pulsed mode. The experimental parameters were strictly controlled: a UV laser pulse repetition rate of 200 kHz, a laser pulse energy of 60 pJ, and a target detection rate of 0.3% (3 ions per 1000 pulses). During data collection, the specimen temperature was maintained at approximately 25 K under an ultra‐high vacuum of 5 × 10^−11^ Torr to minimize thermal migration and surface diffusion. Finally, the 3D reconstruction and compositional analysis of the acquired data were performed using the IVAS module within the AP Suite 6.3 software package.

Mechanical properties were characterized through Vickers hardness tests, conducted using a Wilson VH3100 Hardness Tester to evaluate hardness under different aging conditions. Tensile properties were measured at room temperature with a tensile rate of 2 mm∙min^−^^1^, using tensile specimens machined along the rolling direction, with a gauge length of 75 mm and cross‐sectional dimensions of 12.5 × 1.3 mm on the SHIMADZU AGX100KN universal testing machine. For each condition, three tensile tests were performed to obtain average values, ensuring the reliability of the mechanical data.

### 2.3 Model Formulation

The solute distribution in the molten pool was simulated using a continuum formulation based on the Volume‐Averaging Method (VAM). Governed by the conservation of species mass, the continuous solute transport equation accounts for advection in the liquid phase, diffusion in both liquid and solid phases, and the solute rejection at the solid‐liquid interface [25, 26]. The governing equation is expressed as:

(1) $$\begin{array}{cc} f_{l}p_{l}\frac{\partial c_{l}^{m}}{\partial t} & +f_{l}p_{l}\cdot \nabla \;c_{l}^{m}=\nabla \cdot \left(f_{l}p_{l}D_{l}^{m}\nabla c_{l}^{m}\right)+\left(c_{l}^{m}-c_{sl}^{m}\right)\frac{\partial f_{s}p_{s}}{\partial t}\\ & +\frac{Sp_{s}D_{s}^{m}}{l}\left(c_{s}^{m}-c_{sl}^{m}\right) \end{array}$$

where the last term on the right‐hand side represents the solid‐state back‐diffusion, which is critical for high‐alloy systems with wide freezing ranges. Here, *m* is the solute species; *l* = *f_s_d_2_*/6, is the diffusion length; *d_2_* denotes the secondary dendrite arm spacing; *S* = 2/*d_2_* is the interfacial area concentration; and *sl* and *D* correspond to the solid/liquid interface and diffusivity, respectively. Given the sub‐rapid solidification characteristics of the TRC process, local equilibrium at the interface may deviate. Therefore, the dendrite tip growth kinetics were described using the Kurz‐Giovanola‐Trivedi (KGT) model, which links the growth velocity (*v_tip_*) to the local undercooling (Δ*T*) [27]:

(2) $$v_{tip}=C{\left(\Delta T\right)}^{2}+D{\left(\Delta T\right)}^{3}$$

where *C* and *D* are empirical constants.

## 3 Results and Discussion

### 3.1 Achieving Synergistic Improvement in Strength and Toughness Through Cluster Strengthening

**FIGURE 1:**
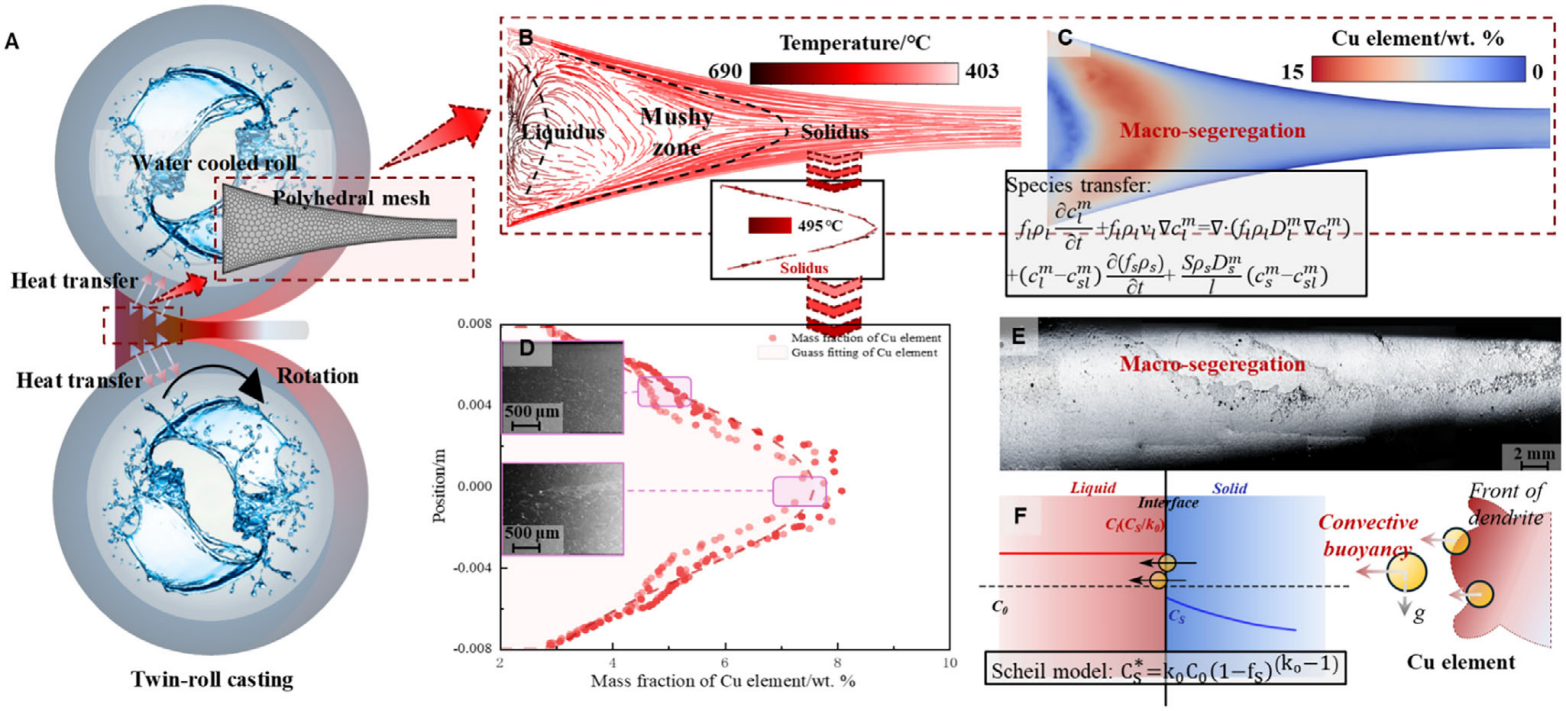
High‐alloy Al‐Li alloy prepared via Twin‐Roll Casting (TRC), a representative sub‐rapid solidification process. (a) The forming zone between the rolls was modeled using a polyhedral mesh, while metal phase transitions and the eutectic phase diagram were defined via a user‐defined function (UDF). A coupled flow field, heat transfer, solidification, and solute transport model was established (B and C). The solute field of Cu at the solidus corresponds to SEM microstructures, showing a clear relationship between the solute field's centerline segregation and the metallographic features of the actual microstructure (D, E). This segregation behavior aligns well with predictions from the Scheil solidification model (F).

In the twin‐roll casting (TRC) process, rapid cooling promotes microstructural refinement at the solid‐liquid interface (Figure 1a). However, such rapid solidification also significantly shortens the time for available solute diffusion at the interface, especially in high‐alloy compositions. This leads to solute accumulation and segregation within the two‐phase region [28]. As alloying elements increase, the solid‐liquid two‐phase region expands considerably, as predicted by equilibrium phase diagrams. Elevated concentrations of alloying elements like Cu and Li extend the freezing range, complicating solute distribution control during solidification [29, 30].

To mitigate the impact of solute accumulation in the two‐phase region, a rigorous optimization of the processing window was conducted, guided by the critical thermo‐mechanical coupling characteristics of high‐alloy systems. A casting speed of 1.05 m/min was adopted in this study, deliberately lower than the conventional range (typically 1.2–1.4 m/min) used for standard aluminum alloys. This specific low‐speed strategy was necessitated by the wide freezing range of the Al‐Cu‐Li alloy. As elucidated in our previous analysis of high‐alloy rheology [31] and parallel studies on complex aluminum systems [28, 29, 30, 31, 32], higher casting speeds excessively deepen the liquid sump, causing the solidification front to approach the roll nip and inducing severe turbulence‐driven segregation. By reducing the speed to 1.05 m/min, the solidification front curvature is effectively flattened, ensuring a stable transition zone and suppressing the stress‐induced flow of solute‐rich liquid responsible for centerline and inverse segregation.

Regarding the rolling mechanics, in situ torque monitoring revealed that a decrease in casting speed correlated with a reduction in rolling resistance. While this observation appears to contradict the theoretical expectation that a lower speed—and the associated thicker solidified shell—would increase deformation resistance, it is attributed to the dominance of strain‐rate and thermal softening mechanisms in this high‐alloy system. Primarily, reducing the speed from 1.4 to 1.05 m/min results in a significant reduction (∼25%) in the mean strain rate. Given the high strain‐rate sensitivity of the semi‐solid/solid alloy at elevated temperatures, this reduction leads to a substantial decrease in flow stress. Concurrently, contrary to simple cooling models, a lower casting speed resulted in a marginally higher strip exit temperature in our specific experimental setup. This phenomenon arises from the saturation of heat flux at the roll‐strip interface; the prolonged contact time reduces the temperature gradient at the interface, diminishing the efficiency of heat removal per unit volume for this high‐latent‐heat alloy. Consequently, the combined effects of rheological softening (due to reduced strain rate) and thermal softening (due to heat retention) effectively override the geometric hardening effect caused by shell thickening, resulting in the observed reduction in rolling load.

Nevertheless, segregation defects were not completely eliminated. To further quantify and control solute migration during solidification, we developed a comprehensive solute field model that incorporated secondary development based on phase diagrams, eutectic transformation, and constitutive behavior of the material, coupled with thermal‐flow‐mechanical conditions (Figure 1b, c). This model enabled the simulation of solute distribution at the solid‐liquid interface, offering a deeper understanding of the nature of segregation. The simulation results revealed significant enrichment of fast‐diffusing solutes, such as Cu, at the interface and their concentration along the centerline, displaying typical macrosegregation characteristics. The predicted distribution pattern was consistent with actual microstructural observations of solute elements (Figure 1d, e). As observed in Figure 1e, slight inverse segregation is present at the sheet edges. Our previous investigations [32, 33] have identified two primary stress‐induced mechanisms for this phenomenon in TRC: the squeezing of solute‐rich liquid from the center to the edges, and the suction of liquid into shear‐induced voids under negative pressure. However, thanks to the optimized low‐speed process adopted in this study, the severity of inverse segregation has been effectively mitigated. Consequently, this study focuses primarily on centerline segregation, which represents the most severe and persistent heterogeneity in high‐alloy TRC sheets, serving as the critical target for evaluating the efficacy of the proposed cluster‐mediated strengthening strategy.

This solute accumulation in the liquid phase aligns with the Scheil solidification model, which describes solute distribution when the partition coefficient *k_0_* <1 (Figure 1f) [34]:

(3) $$C_{s}^{*}=k_{0}\;C_{0}{\left(1-f_{s}\right)}^{k_{0}-1}$$

where$\;C_{s}^{*}$ represents the solute concentration at the interface, *C*_0_ is the original concentration, and *f_s_* is the fraction of solid phase. During solidification, Cu migrates toward the interface as a result of solutal buoyancy. The degree of segregation was further quantified by extracting solute concentrations at the mushy zone boundary and calculating the segregation ratio (*K*) using the following formula:

(4) $$K=\frac{C_{s}}{C_{0}}\;$$

where *C_s_* is the solute concentration in the solid phase. The results showed a segregation ratio of 2.06 at the center, indicating positive segregation, and 0.80 at the edge, indicating negative segregation, with simulation results showing less than 5% deviation from experimental compositional measurements. These findings highlight the high consistency between simulation and experimental data.

The mushy zone, characterized by solute accumulation, can be conceptualized as an incompressible “saturated sponge,” comprising a solid skeleton and a liquid‐filled porous network. Under the compressive forces exerted by the rolls during casting, the solid skeleton expels solute‐rich liquid. Previous studies confirmed the existence of a stress field analogous to the rolling process within the TRC system. When the solute‐enriched liquid is squeezed along the rolling direction, the solid phase, rapidly flowing toward the casting outlet, entrains this liquid, thereby forming large‐scale macrosegregation zones in the sheet. This is evident in Figure 1e, which illustrates the flow‐induced segregation pattern, explaining why high‐solute segregation persists even under high cooling rates in the TRC process.

The convective behaviors observed in Figure 1b, c, corroborated by theoretical solidification studies, further support this phenomenon. In the two‐phase region, the flow field demonstrates rapid changes in flow patterns and alternating convective heat transfer, while the solute field exhibits substantial enrichment and frequent fluctuations. Such segregation directly affects the distribution of precipitates, ultimately impacting the overall material performance.

**FIGURE 2:**
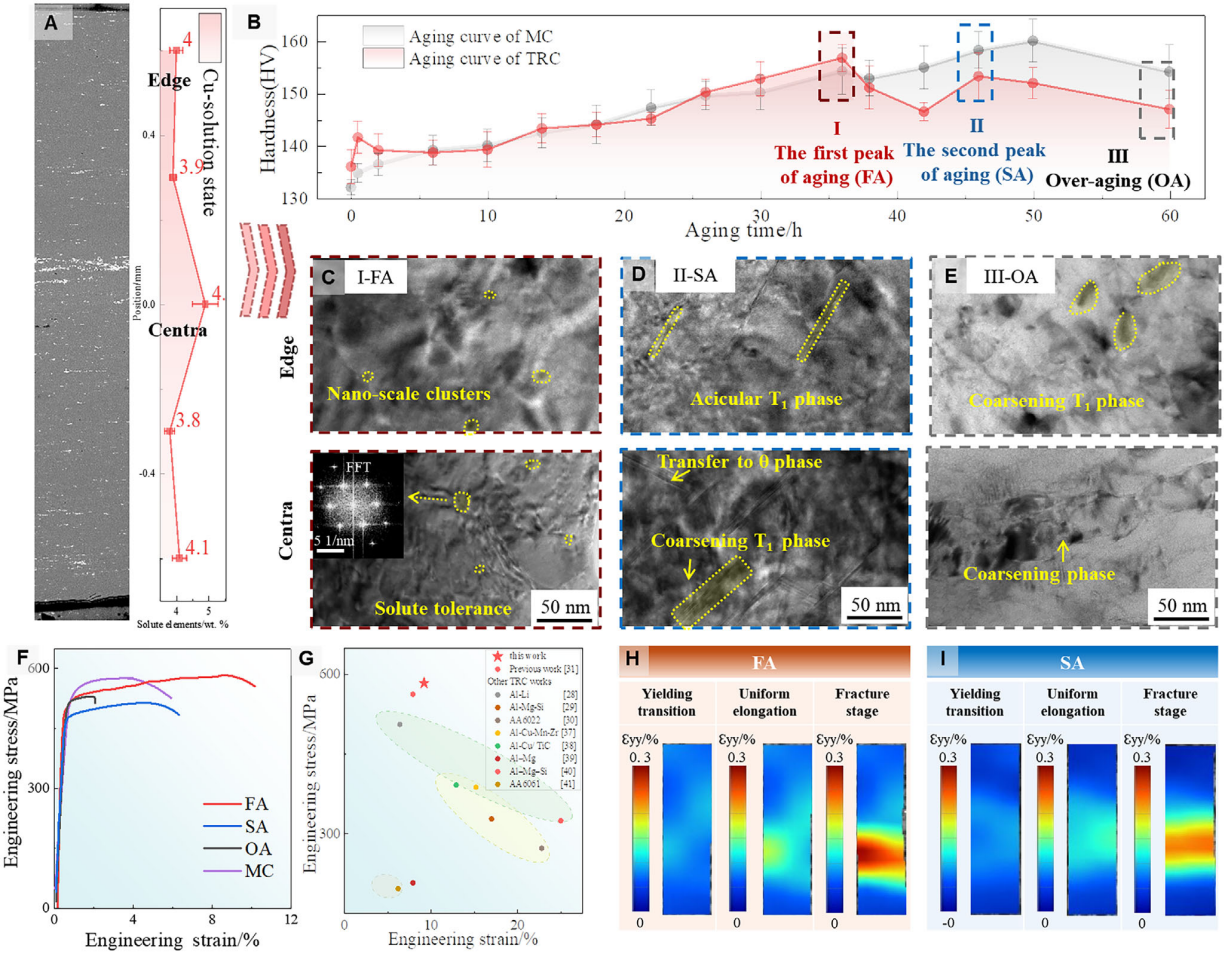
Evolution of microstructure and solute segregation in TRC samples during homogenization and aging. Despite rolling, solution treatment, and quenching, macrosegregation of Cu still remain, as shown by SEM analysis (A). Hardness curves for MC and TRC samples aged for 0–60 h (B) revealed a distinct double‐peak in the segregated microstructure, corresponding to the cluster‐dominated FA stage, needle‐like T1 phase in SA, and over‐aged OA state (C–E). Tensile properties under different aging conditions (F) highlighted the cluster‐strengthened samples, which achieved international‐level mechanical properties (G). Deformation in FA and SA tensile samples was observed in situ using DIC (H, I).

Although post‐homogenization and rolling processes alleviate some of the solute heterogeneity, residual compositional gradients still remain. After solution treatment, the segregation ratio decreases to 1.24 in the central region and to 0.96 at the edge, showing a similar trend to the as‐cast state, albeit with reduced severity. However, macrosegregation transformed into localized microsegregation, as evidenced by the formation of coarse intergranular phases in Figure 2a, a phenomenon also reported in previous studies [35].

This compositional heterogeneity further leads to anomalous aging behavior in the hardness curve. In traditional Al‐Li alloys, the aging curve typically exhibits a single peak during T1 phase precipitation. However, in segregation‐sensitive microstructures, the aging curve displayed an unusual double‐peak characteristic (Figure 2b), with the first peak at 35 h showing greater strength than the second peak at 46 h. TEM analysis of the samples taken from the first peak‐aged (FA), second peak‐aged (SA), and over‐aged (OA) conditions revealed that the FA sample predominantly consisted of clusters, while the SA sample was characterized by T1 phases, and the OA sample exhibited coarsened T1 phases. In this study, clusters are defined as coherent, solute‐enriched aggregates that possess short‐range order but lack the defined long‐range crystallographic structure of equilibrium precipitates. Distinguishing these clusters from fine precipitates was achieved through Fast Fourier Transform (FFT) analysis and morphological assessment. While crystalline precipitates typically exhibit distinct, sharp superlattice reflection spots in FFT patterns, the clusters identified here manifest only as diffuse scattering or weak streaks without extra diffraction spots, as shown in Figure 2c, indicating the absence of a periodic lattice structure distinct from the matrix. It should be noted that the dark features observed in the bright‐field TEM images (Figure 2c) appear larger than typical atomic clusters. This discrepancy is attributed to coherency strain contrast: the lattice mismatch between the coherent clusters and the matrix generates a strain field that scatters electrons, creating a contrast region significantly larger than the physical size of the solute aggregate. To definitively validate the nature of these features, APT analysis was conducted on the same FA state (see Figure 7). The APT data confirms that the microstructure is indeed dominated by high‐density solute clusters with. Furthermore, the FFT patterns in Figure 2c show characteristic diffuse scattering absent of sharp superlattice spots, ruling out the presence of well‐developed precipitates. Thus, the features in Figure 2 are identified as coherency strain fields induced by the dense population of solute clusters.

In FA samples, clusters exhibited high solute tolerance. In the solute‐rich central region, clusters slightly increased in size and density, while maintaining structural stability; in the solute‐poor edge region, clusters showed a more dispersed distribution (Figure 2c). In contrast, SA samples exhibited coarsening or phase transformation of needle‐like precipitates in solute‐rich regions, reducing the strengthening contribution of these phases. This transformation of T1 into θ' phase in Cu‐rich regions has been observed and discussed in detail in Li's study [28]. In the solute‐poor regions, the number density of T1 precipitates was minimal, indicating high instability.

The compositional tolerance of clusters is attributed to their nanoscale dimensions, which enable localized redistribution and adjustment of solute elements. Liu's research [23, 24] demonstrated that nanoclusters possess a low interfacial energy in the aluminum matrix, promoting self‐regulation and effective absorption of solute atoms. This nanoscale adjustment capability makes clusters less sensitive to compositional variations, offering a significant advantage in high‐alloy systems.

More importantly, in segregation‐sensitive processing, the FA samples exhibited a 5.1% increase in HV hardness compared to the SA samples, highlighting the potential of cluster‐dominated strengthening for property enhancement. Figure 2f presents the tensile curves for TRC‐produced Al‐Li alloys under different precipitation states. Consistent with the hardness results, the FA samples reached a tensile strength of 584.0 MPa and an elongation of 9.2%, representing an 18.7% and 41.5% improvement over the SA samples, respectively. This confirms that the cluster‐dominated state achieves a synergistic enhancement in both strength and ductility.

As shown in Figure 2f, the MC samples, benefiting from a homogeneous initial microstructure, exhibited a high ultimate tensile strength of 576 ± 5 MPa, as shown in Table 1. Remarkably, despite the inherent segregation heredity in TRC, the cluster‐dominated FA samples achieved a strength level comparable to this MC benchmark. Mechanistically, this performance defies the limitations typically imposed by the complex thermo‐mechanical coupling of the TRC process. While the sub‐rapid cooling rate inherently promotes solute trapping, the mechanical rolling force imposed on the mushy zone acts as a counteracting factor, driving solute‐rich liquid through interdendritic channels and exacerbating macro‐segregation. However, the high solute tolerance of nanoclusters effectively compensates for this lack of microstructural uniformity. By leveraging the cluster‐dominated state, the material accommodates these inherent segregations, maintaining a robust balance of strength and ductility.

**TABLE 1:** Mechanical properties of the TRC 2060 alloys compared with MC.

Sample State	Yield Strength(σ_0.2_, MPa)	Ultimate Tensile Strength (σ_b_, MPa)	Elongation (δ, %)
FA	519.2 ± 5.5	584.0 ± 10.2	9.2 ± 0.5
SA	445.6 ± 10.1	492.1 ± 15.8	6.5 ±0.4
OA	515.3 ± 11.2	535.4 ± 16.5	2.5 ± 0.3
MC	524.0 ± 4.4	576.0 ± 5.0	6.0 ± 0.4

It is also noted that the OA samples exhibited a slightly higher yield strength than the SA samples. This is attributed to the Orowan bypass mechanism governing the over‐aged state: while the coarsening of precipitates reduces the obstacle density, the coarse non‐shearable particles maintain a high critical stress for the onset of dislocation motion [36].

Further understanding was gained via digital image correlation (DIC) analysis of the strain distribution (Figure 2h, i). Compared to the uniform deformation observed in FA samples, the SA samples exhibited a more heterogeneous strain distribution, attributed to macrosegregation‐induced disparities in T1 phase precipitation. In SA samples, local strain concentrations rapidly developed in low‐resistance regions, leading to premature fracture. In contrast, the effective mitigation of segregation effects in FA samples promoted uniform strain accommodation and improved toughness.

Based on these synergistic mechanisms, the TRC‐produced Al‐Li alloy under cluster‐strengthening strategies demonstrated significant performance improvements, ultimately reaching internationally leading standards for Al‐Li alloys as illustrated in Figure 2g [37, 38, 39, 40, 41]. This comparison highlights that the precise regulation of cluster formation unlocks the potential of sub‐rapid solidification. This strategy offers a promising pathway to balance the performance and production efficiency of high‐alloy Al‐Li alloys, suggesting their potential for application in high‐demand fields such as aerospace.

### 3.2 Enhanced Plasticity Through Multi‐Dislocation Activation Mechanisms

Nanoscale clusters formed in the segregation‐prone microstructure significantly enhance the strength and toughness of high‐alloy Al‐Li systems, but their effects on plastic deformation require a thorough discussion. During aging and tensile testing of aluminum alloys, lower‐temperature aging provides limited energy that primarily affects nanoscale precipitate transformations without significantly altering dislocation density or grain orientation at the macro level [42]. Thus, it is reasonable to assume that the microstructures of FA and SA samples are essentially similar before tensile deformation.

**FIGURE 3:**
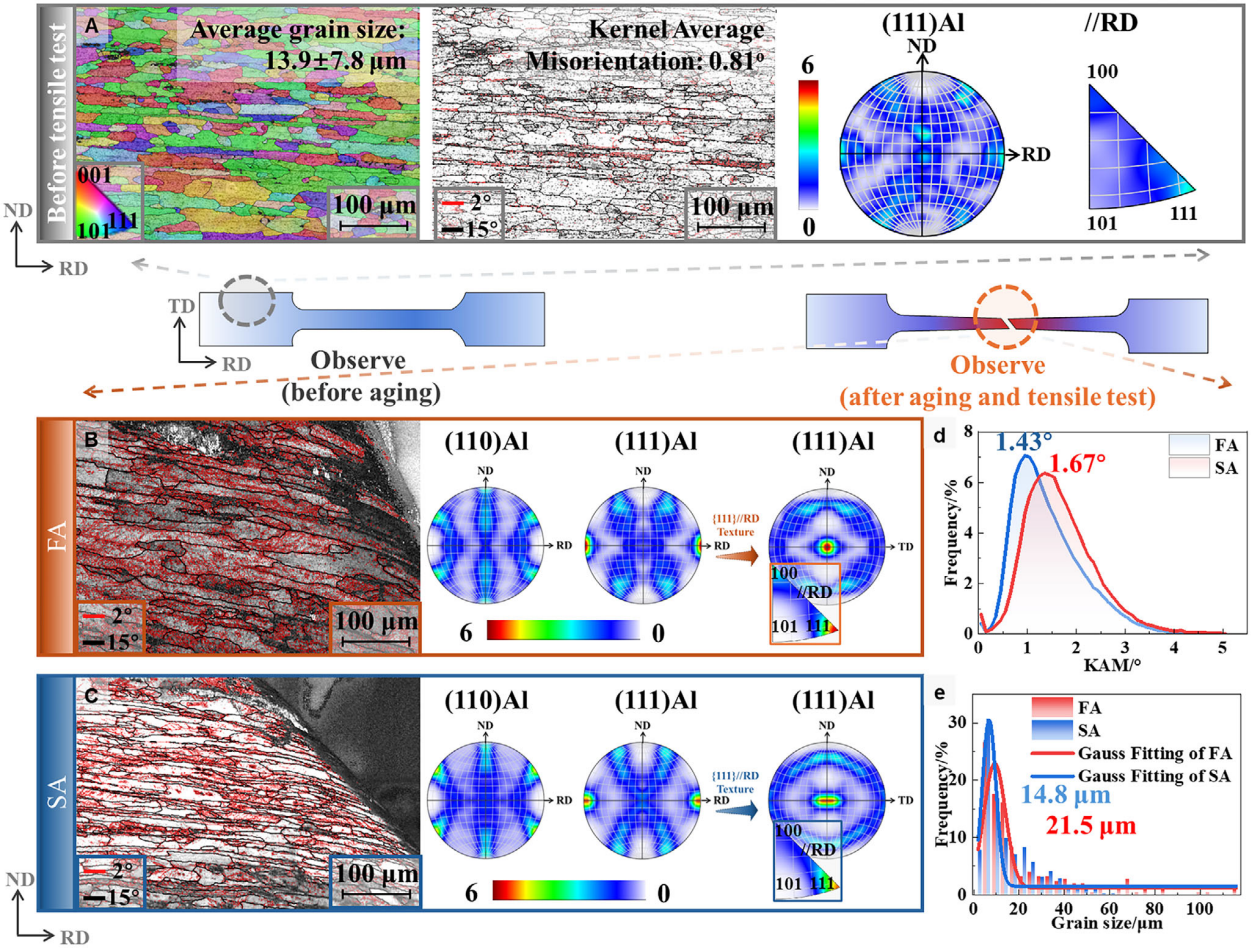
EBSD analysis of microstructure before and after tensile testing. Inverse pole figure (IPF), grain boundary map, and pole figure of the microstructure prior to tensile deformation were analyzed using EBSD (A). Post‐tensile fracture analysis for the first peak‐aged (FA) (B) and second peak‐aged (SA) (C) samples shows grain boundary distributions and corresponding pole figures. Kernel Average Misorientation (KAM) distributions (D) and grain size statistics (E) for the fractured samples were also compared, showing distinctions between the two aging states.

Figure 3 shows EBSD characterization of FA and SA samples before and after tensile testing, with the tensile load applied parallel to the rolling and casting directions.

Figure 3a illustrates the initial microstructure, including the IPF map, KAM map, (111) pole figure, and inverse pole figure. Due to the T8 pre‐deformation process, deformation grains oriented along the RD were observed, and the dislocation density reached 0.81°. Additionally, a minor <111> tensile deformation texture was present.

After tensile testing, FA samples exhibited more pronounced plastic deformation than SA samples, particularly in the necking regions (Figure 3b, c). The average equivalent grain diameter in FA samples increased to 21.5 µm, which is 45.3% larger compared to SA samples and 54.7% larger compared to the undeformed microstructure (Figure 3d). KAM values also increased significantly to 1.67°, indicating higher dislocation accumulation in FA compared to SA (Figure 3e). Grain deformation after tensile testing directly reflects plastic deformation before fracture. Larger post‐deformation grain sizes in initially similar samples indicate higher plasticity and better post‐yield toughness.

The plastic deformation also enhanced texture alignment. Both FA and SA samples showed RD‐aligned fiber textures along the (111) plane, characterized by load axis poles surrounded by annular texture patterns that were recognizable in the ND‐TD plane section. As deformation progressed, dislocation glide and multiplication along the <111> direction in the FCC grains caused deformation and rotation, leading to a developed <111> texture. Moreover, SA samples exhibited stronger RD fiber texture and <111> alignment, suggesting excellent synergistic deformability.

**FIGURE 4:**
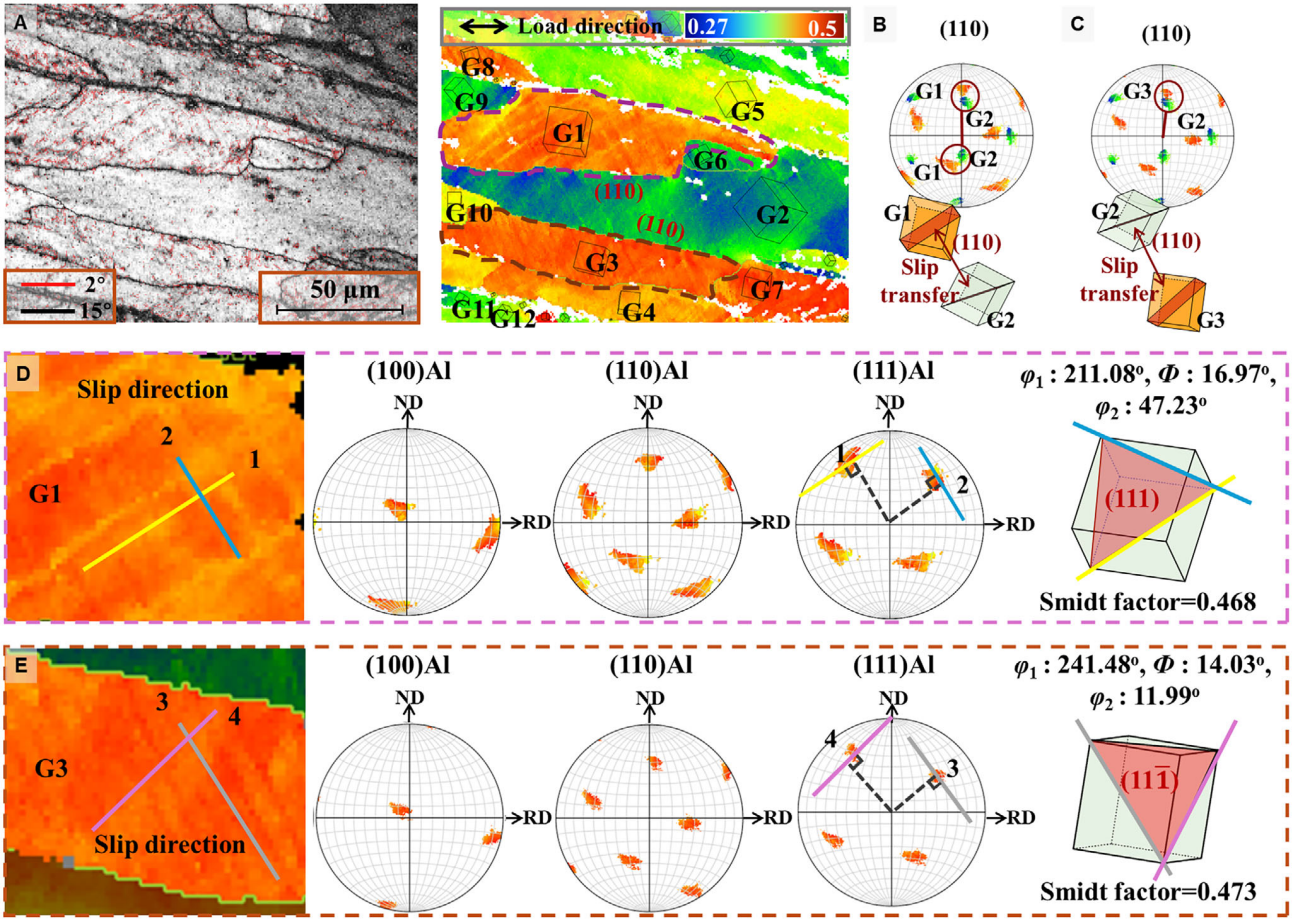
EBSD analysis of high‐deformation grain regions in the SA sample. High‐magnification analysis of grain boundaries and Schmid factors was performed on the severely deformed regions (A). Dislocation transmission across the grain boundaries of G1 and G2 was analyzed using pole figures (B), as well as transmission between G2 and G3 (C). Slip traces and grain orientation were used to study the slip activity and lower dislocation activation resistance in G1 (D) and G3 (E).

Further analysis of dislocation slip behavior in SA samples was conducted using high‐resolution EBSD near the fracture surface (Figure 4a). The grain boundary and Schmid factor distributions revealed significant slip traces. The Schmid factor, which is inversely related to critical shear stress [43], quantifies the ease of activating a slip system. In the SA samples, grains such as G1 and G3 exhibited high Schmid factors, leading to early activation of slip systems. Conversely, Bai's in situ EBSD study [44] indicated that deformation potential decreases as slip progresses, explaining the reason behind the significant deformation in certain grains, such as G2, despite their low Schmid factor.

The evaluation of deformation also depends on the alignment of slip systems across grain boundaries, which determines whether dislocations can be transmitted, thereby releasing stress concentration. The nearly parallel slip planes of grains G1‐G3 provided favorable conditions for dislocation transfer, resulting in aligned grain boundaries without stress concentration features after deformation. More importantly, Figure 4b, c demonstrates that the G1‐G2 and G2‐G3 grains shared a common grain boundary in the (110) plane, which further facilitated the transfer of slip.

Plastic deformation also depends on dislocation multiplication and motion within grains. Figure 4d, e shows abundant slip traces in two directions within G1 and G3. These traces were found to be perpendicular to lines originating from the (111) plane in the pole figure, indicating dislocation slip along the {111} plane family. Euler angle fitting revealed multi‐slip activation: (111)<110> and (111)< 01$\bar{1}$> in G1, and (11$\bar{1}$)< 10$\bar{1}$> and (11$\bar{1}$)< 0$\bar{1}$1> in G2. This multi‐dislocation activation facilitated coordinated slip, significantly enhancing the alloy's plasticity.

The improved plasticity in FA samples is primarily due to the reduced activation energy (*E_act_*) for dislocation movement in clusters, compared to the needle‐like phases. This relationship can be expressed as:

(5) $$E_{act}\propto \tau \cdot V_{act}$$

where τ is the applied stress and *V_act_* is the activation volume. Under identical stress conditions, the activation volume of a cluster and a needle‐like precipitate can be compared using simplified calculations. For clusters, assuming α solute atoms with a per‐atom volume *V_cluster_*:

(6) $$V_{cluster}=\frac{4}{3}\pi {\left(\alpha \cdot r_{cluster}\right)}^{3}$$

where α is a material−specific constant related to elasticity and stress, typically between 2 and 4 [45], and *r_cluster_* is the cluster radius. Without atomic forces:

(7) $$Nv=\frac{4}{3}\pi {r_{cluster}}^{3}$$

For needle‐like precipitates:

(8) $$V_{needle}=\pi {r_{needle}}^{2}l_{needle}+2\cdot {\left(\beta \cdot l_{needle}\right)}^{3}$$

(9) $$Nv=\pi {r_{needle}}^{2}l_{needle}$$

where β is typically between 1 and 3 [46]; *l_needle_* and *r_cluster_* are the length and radius of the needle‐like precipitate, respectively. Assuming ɑ and β take minimum values, we find:

(10) $${l_{cluster}}^{3}>14.7{r_{cluster}}^{3}$$

Given that the radius of a needle‐like precipitate is 3–5 atomic diameters and its length‐to‐radius ratio ranges from 100 to 1000, while the cluster is nearly spherical with a ratio close to unity, this inequality holds valid. Thus, clusters are more likely to act as initiation sites for dislocation multiplication, promoting cooperative motion and enhancing plasticity.

It is important to acknowledge the geometric limitations of the current model. Equations (5)—(10) utilize a spherical assumption for nanoclusters to facilitate the analytical derivation of the critical shear‐bypass transition size. In reality, solute clusters may exhibit irregular morphologies or adopt specific crystallographic orientations due to lattice misfit strains, as noted in previous studies [47]. However, the spherical model serves as a standard equivalent volume approximation, allowing for the conversion of complex 3D solute aggregates into a manageable 1D parameter (equivalent radius). Therefore, while this assumption introduces some geometric simplification, it provides a reasonable first‐order estimation of the strengthening mechanisms without the excessive complexity of shape factors.

The interaction between dislocations and clusters provides a balance between strength and ductility, which will be explored in the next section.

### 3.3 Strength Enhancement Through a Shear‐Bypass Synergistic Strengthening Mechanisms

**FIGURE 5:**
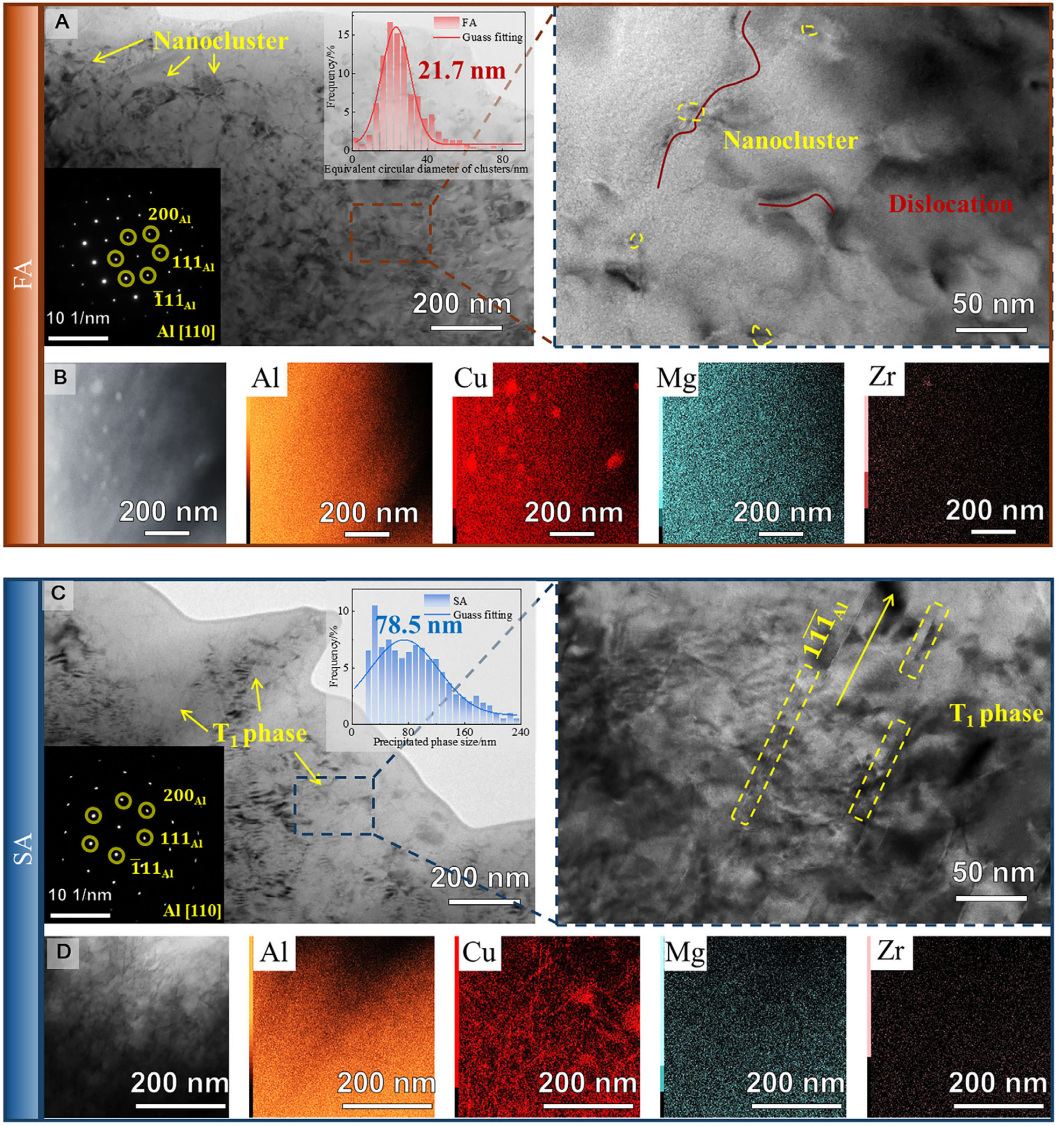
TEM analysis of the precipitate morphology after tensile deformation. The precipitate morphology in FA (A) and SA (C) samples was observed at both low and high magnifications, and the size of the precipitates was statistically analyzed. Elemental distribution mapping of FA (B) and SA (D) samples was performed through EDS to visualize the distribution of key alloying elements.

The initial microstructure, processing conditions, and solution treatment of FA and SA samples were identical, suggesting that their strength differences primarily arise from variations in precipitate characteristics. Their pre‐deformation microstructure has been outlined in Figure 2, with detailed discussions provided in the Supporting Information. To explore dislocation‐precipitate interaction mechanisms, TEM characterization was conducted on fracture regions of both FA and SA samples (Figure 5), revealing marked differences.

In the FA samples, a high density of fine nanoscale clusters was observed, as shown in the updated Figure 5a. These clusters are fully coherent with the matrix and exhibit an average equivalent diameter of ∼3.5 nm, which aligns well with the atomic‐scale observations from APT (2–15 nm) discussed later. This indicates a uniform and dispersed distribution of strengthening units throughout the microstructure. In contrast, SA samples were predominantly characterized by needle‐like T1 precipitates, with an average length of 78.5 nm and a standard deviation of 96.2 nm, indicating considerable variation in size. Additionally, large‐scale precipitates formed due to compositional instability were observed in SA. As noted by Li et al. [48], the segregation‐prone microstructure in Al‐Li alloys tends to form θ' or δ' phases, which can significantly degrade strengthening efficacy.

The distribution differences between precipitates in FA and SA were further confirmed through EDS mapping (Figure 5b, d). In FA samples, Cu was uniformly dispersed, with free Cu atoms effectively incorporated into clusters to form strengthening phases. The strong correspondence between Cu and Mg distribution in FA indicates that clusters have a high solute tolerance, accommodating elements such as Cu and Mg effectively, thereby regulating segregation. In contrast, SA samples exhibited inhomogeneous Cu distributions, forming needle‐like T1, spherical clusters, or even large multi‐layered T1 precipitates, leading to pronounced segregation and reduced strengthening efficiency—a key reason for the performance decay observed in SA. The size statistics of the precipitates, when input into Equation (8), still satisfy the previously derived inequalities, thereby reinforcing our earlier conclusions.

**FIGURE 6:**
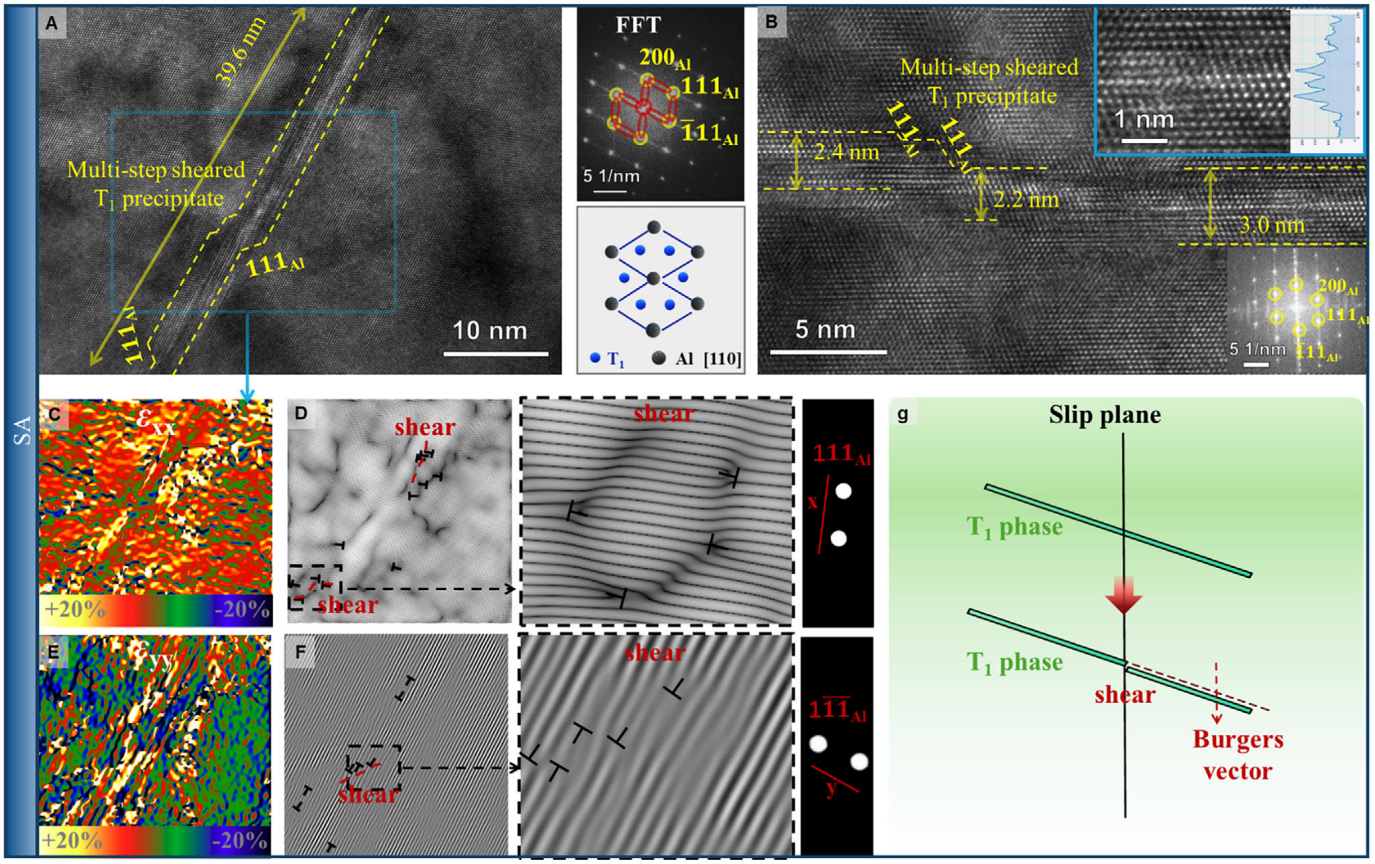
HRTEM analysis of dislocation shearing through the T1 phase. In the SA sample, the T1 phase was identified via diffraction spots in the FFT map, and dislocation shearing was observed (A). The shear dimension was measured (B). Stress fields along the <111> direction perpendicular to the T1 phase were plotted (C), and the corresponding IFFT transformation images were depicted (D). Similarly, stress fields and IFFT transformation images along the <1$\bar{1}\bar{1}$> growth direction of the T1 phase were presented (E, F). Dislocation behavior during shearing through the T1 phase was further analyzed (G).

To better understand the strengthening behavior, we first analyzed the traditional dislocation‐precipitate interaction mechanism for the T1 phase in SA, serving as a baseline. High‐resolution TEM (HRTEM) images from SA samples, combined with Fast Fourier Transform (FFT) analysis, confirmed that the precipitates observed in Figure 6a were T1 phases. These T1 phases, characterized by a coarse morphology, were ideal for analysis in this study. Figure 6b shows typical shearing behavior across T1 precipitates at thicknesses of 2.4, 2.1, and 3.0 nm, where the shearing takes place along the <111> directions in a stepped pattern. Notably, shearing continued even at a thickness of 3.0 nm, suggesting that shearing was the primary mechanism responsible for the strengthening.

Stress field analysis perpendicular to the <111> direction in T1 confirmed high stress concentrations at the shear interface. Inverse FFT (IFFT) analysis of the <111> shear zones revealed the formation of numerous half atomic planes at these interfaces—a signature characteristic of edge dislocations. Similar results were seen in the IFFT along directions parallel to T1 growth, showing dislocations at shear locations. These observations underscore the critical role of dislocation shearing in contributing to material strengthening. Moreover, the interaction between T1 and dislocations induced changes in interfacial energy, stacking fault energy, and strain energy—all of which also contributed to strength [49]. The strengthening contribution due to T1 shearing, σ_Shear_, can be estimated using the following equations:

(11) $$\sigma_{\mathrm{Shear}}=K_{T1}\;D^{2}N^{\frac{1}{2}}t^{-\frac{3}{2}}$$

(12) $$K_{T1}=1.211{r_{eff}}^{\frac{3}{2}}\sqrt{\frac{\pi }{2Gb}}$$

where *D*, *N*, and *t* represent the diameter (78.5 nm), number density (5.2 × 10^21^ m^3^), and thickness (1.95 nm) of the T1 precipitates, respectively; *K*_*T*1_ accounts for stacking fault energy (*r_eff_* = 0.09 J/m^2^), shear modulus (*G* = 27 GPa), and Burgers vector (*b* = 0.286 nm). Calculations estimate the shear strengthening contribution of T1 to be 80.2 MPa.

**FIGURE 7:**
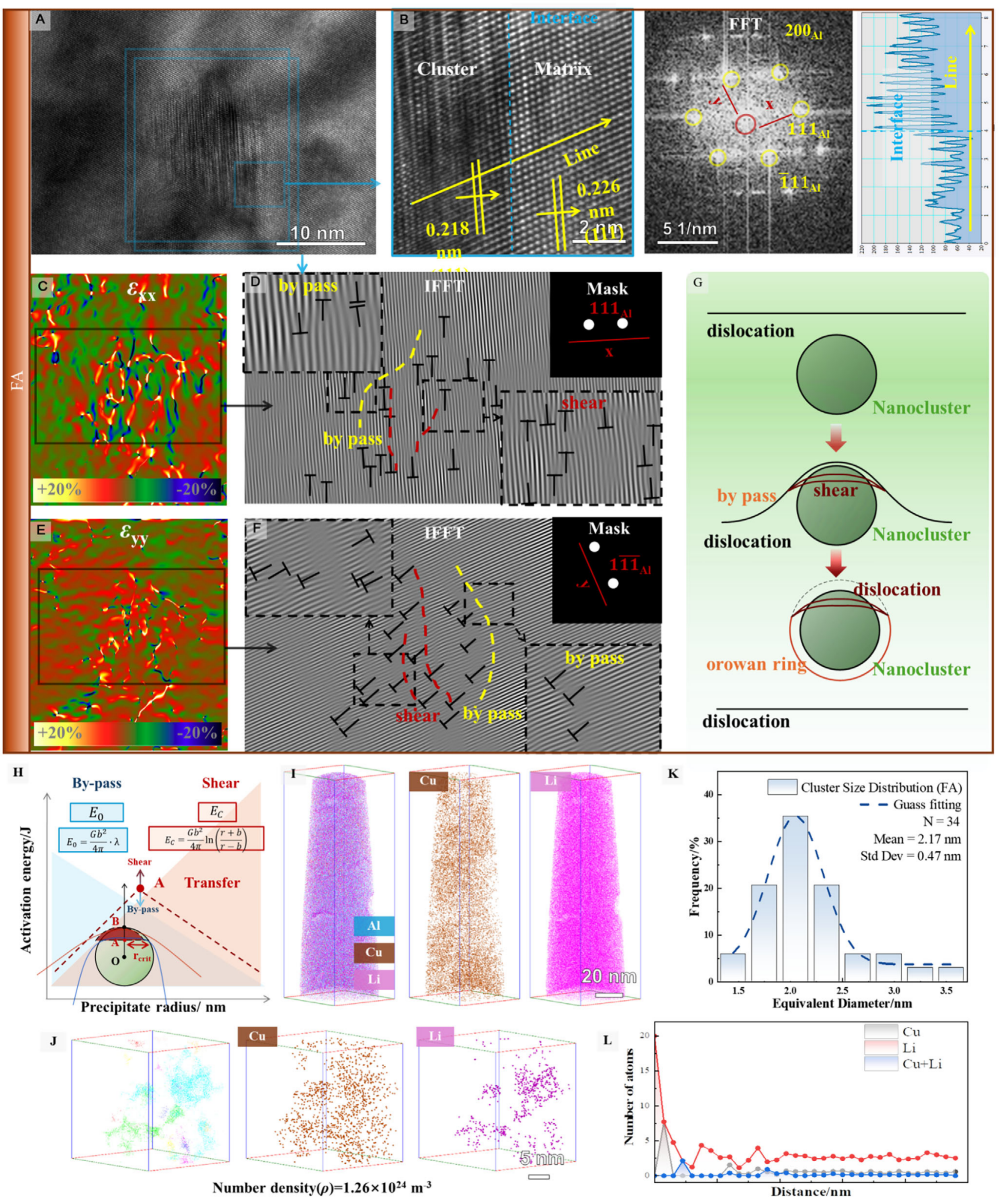
HRTEM analysis of dislocation interaction with nanoclusters. The size of nanoclusters in the FA sample was measured (A), and the interface between clusters and the aluminum matrix was observed, with diffraction spots of clusters identified in the FFT map (B). The shear dimension was measured (B). Stress fields along the <111> direction were plotted (C), along with the corresponding IFFT transformation image (D). Stress fields along the <1$\bar{1}\bar{1}$> direction and their IFFT transformation image were also presented (E, F). Evidence of both dislocation shearing and bypassing nanoclusters was found (G). The shear‐bypass synergistic strengthening mechanism was discussed in detail (H). Atomic‐scale characterization of clusters in FA sample by atom probe tomography (APT) (I), and selective elemental distribution of Cu/Li within and around nanoclusters (J). Statistical size distribution of nanoclusters (K), and quantitative analysis of cluster composition at the atomic scale (L).

The FA samples, containing nanoscale clusters, exhibited complex dislocation‐cluster interactions, involving both bypass and shear mechanisms (Figure 7a). HRTEM images in Figure 7 offer a detailed view of dislocation behavior through typical clusters, which have an interface parallel to the aluminum matrix's (111) plane. The atomic plane spacing within the clusters was 0.218 nm, whereas the Al matrix spacing was 0.226 nm (Figure 7b), resulting in a 3.54% mismatch—indicative of a coherent phase. This coherent interface reduces interfacial energy, preserving lattice coherence and effectively hindering dislocation transmission. Unlike T1 precipitates, which exhibited localized stress concentrations at the cutting sites, clusters displayed uniform stress throughout, ensuring consistent resistance to dislocation motion (Figure 7c, e).

Clusters exhibited tighter atomic plane spacing compared to T1 precipitates due to their nature as solute atom aggregations driven by vacancy concentration and solute‐vacancy bonding interactions. This denser atomic arrangement provides higher resistance to dislocation movement, significantly increasing yield strength.

Additionally, IFFT analysis of <111> and <$1\bar{1}\bar{1}$> direction diffraction spots (Figure 7d, f) showed distinctive features compared to conventional Orowan looping observed with spherical precipitates. Unlike typical Orowan strengthening, where dislocation loops form around precipitates, clusters in FA samples demonstrated mixed behavior: dislocations formed loops not only around the clusters but also bands within them—a hallmark of shearing. This implies that dislocations engage in both bypassing and shearing as they interact with clusters. Strengthening behavior involves an initial shearing, followed by Orowan looping as contact volume and increasing dislocation pile‐up, ultimately maximizing strengthening (Figure 7g). This dynamic interaction, termed shear‐bypass synergistic strengthening, effectively balances strength and ductility. This phenomenon was previously observed in Ni‐based alloys by Li [50] and is here reported for Al‐Li alloys, providing a strengthening strategy for high‐strength aluminum alloys.

There is an intrinsic link between shearing and bypassing mechanisms. Liu's [17] research previously identified a thermodynamic‐critical point dictating dislocation‐precipitate interaction. Thermodynamic insights suggest that this transition occurs when the energy barriers for shearing and bypassing equilibrate. As shown in Figure 7h, at the initial dislocation‐cluster contact phase (AB stage), the shear energy barrier (*E*_S_) is lower, and the bypass barrier (*E*_O_) is higher. As shearing progresses, the contact radius increases, and *E*_S_ eventually matches *E*_O_, triggering a transition at a critical radius *r*_crit_. The activation energy (Δ*E*) can be expressed as:

(13) $$\Delta E=V_{eff}\;\sigma_{\mathrm{strenth}}$$

where *V_eff_* and σ_*strent*h_ represent the effective activation volume and the strengthening stress, respectively. When the energy barriers for shearing and bypassing are equal:

(14) $$E_{S}=E_{O}\;$$

(15) $$E_{S}=AV_{S}\sigma_{S},E_{O}=BV_{O}\sigma_{O}$$

where A and B are constants for energy barrier equality. The shear strength σ_S_ is given by:

(16) $$\sigma_{S}=K_{T1}\;{r_{crit}}^{2}N^{\frac{1}{2}}h^{-\frac{3}{2}}$$

The equivalent spherical cap height (*h*) and volume (*V_S_*) are calculated as:

(17) $$h=r-\sqrt{r^{2}-{r_{crit}}^{2}}$$

(18) $$V_{S}=\frac{\pi h^{2}}{3}\times \left(3r-h\right)$$

The bypass strength (σ_*O*_) and volume (*V_O_*) are described as:

(19) $$\sigma_{O}=\frac{\mathrm{Gb}}{\lambda }\times \ln\left(\frac{r}{b}\right)\cdot \frac{V_{O}}{V_{\mathrm{total}}}$$

(20) $$V_{total}=\frac{4}{3}\pi r^{3},V_{O}=V_{total}\;-V_{S}$$

Using Python‐based fsolve methods, the critical radius was found to be 1.6 nm. In the model, the constants used include *λ* = 3.05 × 10^−8^ m for precipitate spacing, *r* = 3.5 × 10^−9^ m for average cluster radius, *N* = 7.5 × 10^22^ m^−3^ for number density, respectively. These parameters are based on the statistics of the TEM images. Under these conditions, dislocation behavior transitions from shearing to bypassing (Figure 7i). The shear strengthening contribution was calculated as 29.7 MPa, while bypass strengthening contributed 151 MPa, substantially exceeding the strengthening effect of T1 precipitates (80.2 MPa). This difference correlates well with the observed tensile strength difference of 92.4 MPa, demonstrating the effectiveness of the shear‐bypass transition in enhancing material strength.

To provide a rigorous quantitative assessment of the strengthening phase and refine these microstructural parameters, a comprehensive statistical analysis was performed based on the APT reconstruction of the FA sample. For this purpose, a representative sub‐volume of 30 × 30 × 30 nm^3^ was precisely selected within the grain interior away from major segregation bands. While the average cluster diameter is small, this selected volume dimension is sufficient to capture a statistically meaningful population owing to the ultra‐high number density of clusters at this stage. Within this defined volume, 34 individual solute clusters were identified, yielding a calculated number density of approximately 1.26 × 10^24^ m^−3^. The statistical size distribution of these clusters, presented in Figure 7k, shows a narrow, unimodal range with an average equivalent diameter of 2.15 ± 0.49 nm, confirming their fine and uniform dispersion. Furthermore, the average composition profile across the cluster interface was determined using proximity histogram analysis (Figure 7l). The results quantitatively confirm that the cluster cores are significantly enriched in Cu and Mg, with substantial partitioning of Li, proving their nature as multi‐component Cu‐Mg‐Li co‐clusters. This combination of high number density, fine size, and specific composition provides a solid microstructural basis for the exceptional strengthening observed in the FA state.

Consequently, the discrepancy between the apparent TEM feature size (∼2–10 nm) and the precise APT measurement (∼2.15 nm) arises from the projection of a high‐density microstructure through the TEM foil. Statistical overlap of multiple fine clusters along the electron beam creates larger, coalesced features in 2D projections, an artifact eliminated by the atomic‐scale 3D resolution of APT. Crucially, while APT reveals a finer and denser microstructure than initially estimated by TEM, this does not diminish the value of the previously discussed theoretical shear‐to‐bypass transition model. The derived critical transition size holds primarily theoretical significance, aiming to establish the existence and regime of this mechanism rather than providing precise absolute values. Furthermore, the APT results, specifically the elemental distribution showing pronounced Li microsegregation (Figure 7J), experimentally confirm the exceptional solute tolerance of these clusters. This confirms that the clusters can effectively accommodate the localized compositional instabilities induced by the TRC process, supporting the conceptual validity of the proposed strengthening framework.

It is important to clarify that the fundamental dislocation interactions in this study follow classical shear and bypass theories. The concept of leveraging ‘synergetic mechanisms’ to enhance mechanical performance has been explored in other advanced alloy systems; for example, Wang et al. [51] demonstrated the efficacy of synergetic Orowan bypassing and climbing mechanisms in nickel‐base superalloys. Drawing inspiration from such synergistic concepts, this study introduces a distinctive adaptation to the segregation‐prone environment of TRC Al‐Li alloys.

Unlike conventional precipitation strengthening in homogeneous alloys, the cluster‐dominated strategy in this work exploits a dual‐nature advantage to address macroscopic segregation: the coherent clusters are intrinsically shearable, preventing the pile‐up of dislocations and premature cracking at segregation bands; despite their small physical size, the clusters generate extensive coherency strain fields that effectively impede dislocation motion over a large volume.

Therefore, the primary contribution of this work lies not in the discovery of a new physical law, but in the development of a Solute Tolerance Strategy. This approach successfully creates a synergistic equilibrium, enabling the material to accommodate the severe compositional heterogeneity inherited from the TRC process. By harmonizing strength and ductility through cluster‐mediated strengthening, this strategy effectively overcomes a critical bottleneck in the manufacturing of high‐performance Al‐Li sheets.

## 4 Conclusion

Based on the study involving secondary solute field design and nanoscale cluster regulation, the main conclusions of this work are summarized as follows:

1. A shear‐bypass synergistic strengthening mechanism of nanoscale clusters is revealed. This mechanism uniquely combines the advantages of both strengthening modes: the shearing action effectively impedes dislocation motion to enhance strength, while the bypassing action facilitates multi‐directional dislocation passage to accommodate plasticity. This synergy achieves a superior balance, resulting in concurrent enhancements of 18.7% in strength and 41.5% in ductility.
2. Cluster regulation is demonstrated as an effective strategy for mitigating compositional segregation. In the segregation‐sensitive microstructure formed under sub‐rapid solidification, the intentional introduction of nanoscale clusters successfully alleviates the adverse effects of elemental partitioning.
3. The findings provide a technical and theoretical foundation for optimizing the TRC process. The achieved property improvements and the underlying mechanistic understanding offer critical insights for tailoring the sub‐rapid solidification process in twin‐roll casting, supporting the fabrication of high‐performance Al‐Li alloys for demanding aerospace applications.
4. The cluster‐mediated strengthening strategy offers a promising perspective for managing compositional variations in segregation‐sensitive materials. By leveraging the high solute tolerance of nanoclusters, this approach conceptually aligns with the design principles of controlled heterogeneity found in advanced material systems. These findings suggest that similar strategies could potentially be adapted to other compositionally complex alloys to mitigate segregation‐induced performance degradation, although further experimental validation on different alloy systems is required.

## Conflicts of Interest

The authors declare no conflicts of interest.

## Supporting information




**Supporting File 1**: advs74547‐sup‐0001‐SuppMat.docx.

## Data Availability

The data that support the findings of this study are available on request from the corresponding author. The data are not publicly available due to privacy or ethical restrictions.
