# Supplementary material for: Cluster‐Mediated Solute Stabilization and Shear‐Bypass Synergistic Strengthening in High‐Alloyed Systems
Source: Adv Sci (Weinh). 2026 Mar 6;13(25):e22466. doi: 10.1002/advs.202522466 (PMC13137829; doi:10.1002/advs.202522466)
Supplement: Supplementary file 1 — Supporting File 1: advs74547‐sup‐0001‐SuppMat.docx. [file ADVS-13-e22466-s001.docx]

**Supplementary material**

To avoid disrupting the logical flow of the manuscript, detailed descriptions of the model formulation and the associated secondary development work are presented in the **Supplementary Materials**. This includes the formulation of the thermal-flow-solute coupled simulation, the solute field modeling using secondary development.

1. ***Model formulation***


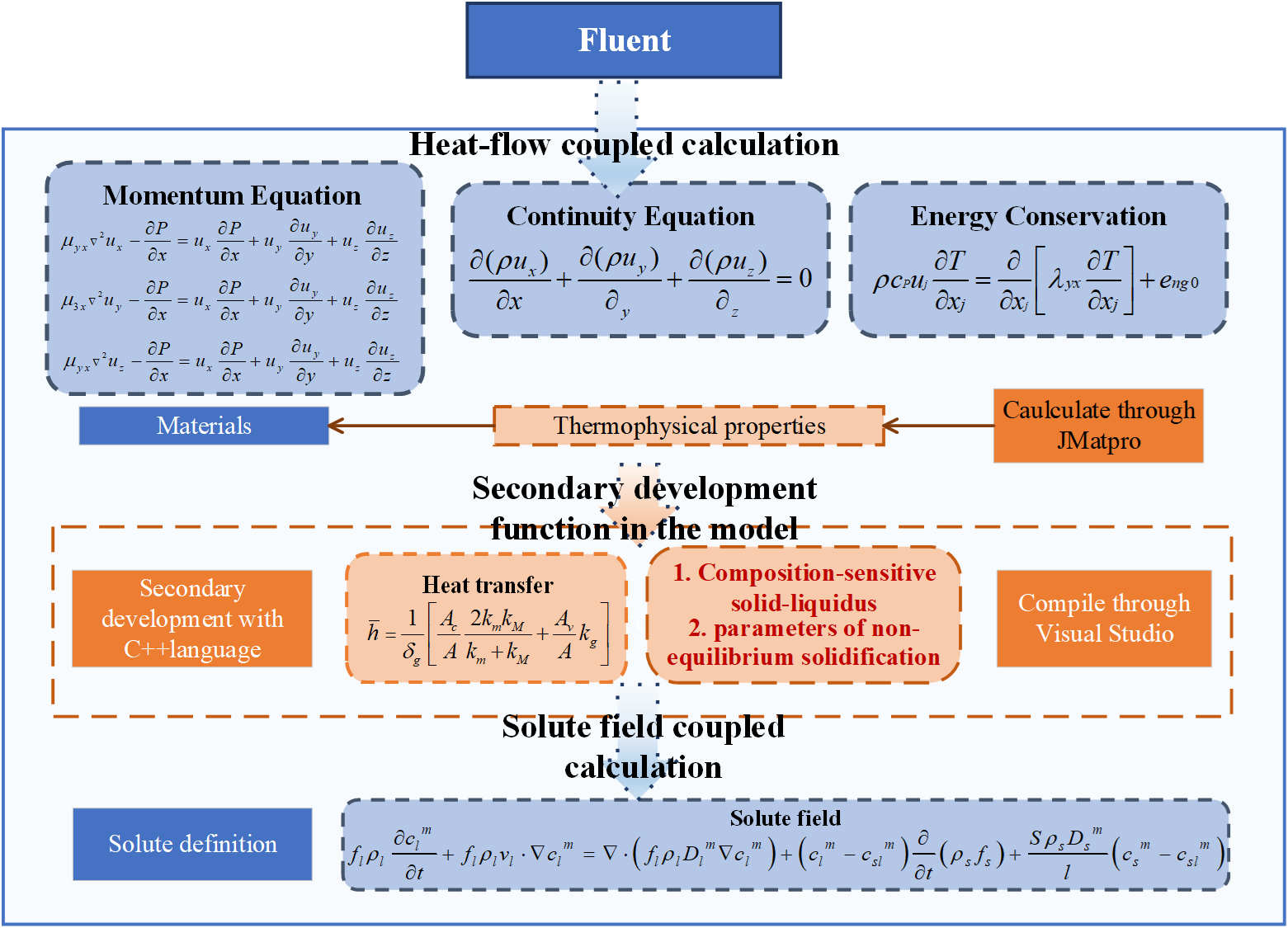


**Fig. S1 Simulation process.** Simulation process for twin-roll casting (TRC) using ANSYS Fluent, enhanced by User Defined Functions (UDF). The model includes momentum, continuity, and energy equations, with secondary development focusing on composition-sensitive equilibrium and non-equilibrium solidification. A solute field was established to represent solute redistribution in high-alloy Al-Li systems.

To simulate the thermal and fluid flow conditions during the twin-roll casting (TRC) process, a detailed model was developed using ANSYS Fluent with supplementary secondary development programming (Fig. S1). The nomenclature and concepts used are summarized in Table S1. The model incorporated Navier-Stokes equations to couple heat transfer and fluid flow in the casting zone. The flow field was represented by a k-ε turbulence model, and heat transfer was modeled using energy conservation principles. Relevant process conditions and material properties are provided in Table S2, while thermophysical properties of the 2060 Al-Li alloy were calculated using JMatPro. The solute transport equation was derived from classical solidification theory, integrating the influences of heat, flow, solidification kinetics, and solute redistribution dynamics. Solute field calculations were performed through a solute transfer model that coupled heat-flow simulation results with a non-equilibrium solidification model. The relevant non-equilibrium parameters are listed in Table S3. This approach ensured a realistic depiction of solute transport, advancing our understanding of solute redistribution in TRC processes for high-alloy Al-Li systems.

Table S1 Main parameters used in simulations of thermal-mechanical coupling in base TRC 2060 Al-Li alloy

|  | Symbol |  | Symbol |
| --- | --- | --- | --- |
| Velocity | *v* | X-direction velocity component | *V_x_* |
| Temperature | *T* | Y-direction velocity component | *V_y_* |
| Pressure | *P* | x coordinate | *x* |
| Time | *t* | y coordinate | *y* |
| Solid-solid contact area | *A_c_* | Effective thermal conductivity of gas | *k_g_* |
| Indirect contact area | *A_v_* | Effective thermal conductivity of roll | *k_M_* |
| Gas film thickness | *δ_g_* | Effective thermal conductivity of melt | *k_m_* |
| Effective thermal conductivity of gas | *k_g_* | Average heat transfer coefficient | $\bar{h}$ |

Table S2 Thermophysical properties of 2060 Al-Li alloy

| Physical parameter | Symbol |  | 30°C | 495°C (solidus) | 648°C (liquidus) | 700°C |
| --- | --- | --- | --- | --- | --- | --- |
| Kinetic viscosity | *μ* | kg/(m·s) | 13.7 | 0.03 | 0.00148 | 0.00125 |
| Specific heat capacity | *C* | J/(kg·°C) | 870 | 1490 | 1070 | 1160 |
| Density | *ρ* | kg/m^3^ | 2790 | 2680 | 2480 | 2460 |
| Thermal conductivity | *k* | W·m | 157.7 | 170.6 | 87.04 | 89.2 |

Table S3 Consider the parameters of non-equilibrium solidification in 2060 Al-Li alloy

| Symbol | Definition and unit | Value |
| --- | --- | --- |
| D(Cu) | Liquid diffusion coefficient of Cu, m²/s | 1.05×10^-7^ exp(-2856/T) |
| D_s_ (Cu) | Solid diffusion coefficient of Cu, m²/s | 4.8×10^-5^ exp(-16069/T) |
| E | Degree of the surface energy anisotropy | 0.02 |
| δ_k_ | Degree of the kinetic anisotropy | 0.3 |
| P | Average Gibbs-Thomson coefficient,m ·K | 1.7×10^-7^ |

Relevant boundary conditions were as follows: the roll radius 250 mm; rotation speed and inlet velocity calculated according to the pull velocity; initial temperature, turbulent intensity, and turbulent viscosity ratio at 690 ℃, 5%, and 10, respectively; pressure at outlet 0 Pa; and the calculation of backflow at this position prohibited. Meanwhile, the grid folding conditions were set at the outlet and the unfolding speed the same as the outlet speed. The folded grid carried the material leaving the casting and rolling area. Gravity applied to the cell zone in the cast-rolling region was (0, -9.81, 0) m/s^2^.

More importantly, some conditions required secondary development process assistance. As solidification progressed, there was an innate velocity decay in the calculation of solid and semi-solid phases. Therefore, it was necessary to define the motion behavior of the solid phase part. The transmission behavior of the solid phase was defined as the arc motion with the central axis symmetrical and the roll center of the corresponding half region as the center, where the corresponding *x*,*y*-direction (S1-2) components were calculated according to the trigonometric function

$\begin{matrix} V_{x}=V\sin\theta=V\sin(\arcsin\frac{-y}{\sqrt{x^{2}+y^{2}}}) \\ V_{\text{y}}=V\cos\theta=V\cos(\arcsin\frac{-y}{\sqrt{x^{2}+y^{2}}}) \end{matrix}$. (S1-2)

Because the transfer equation needed to be calculated in real-time at each location, it needed to be written in C++ language and compiled and run in conjunction with Visual Studio software. Similarly, the heat exchange conditions of TRC were also one of the most important parts of the model and the heat exchange between rolls and casting areas mainly heat conduction and convective heat exchange. The heat transfer process was divided into 5 stages from the inlet (S2), which were the heat transfer coefficient increases as contact progressed. With the thickening of the solidified shell and increased pressure interface contact, the heat transfer coefficient reached a peak. Solidificaion shrinkage, contact separation, heat exchange reduction occurred and, in the cold deformation stage, the sheet cooled, deformation resistance reached a peak, and the high cast-rolling force strengthened the interface contact and enhanced heat exchange. As the temperature further decreased, heat transfer decreased. The process was described as

$\bar{h}=\frac{1}{\delta_{g}}\left[\frac{A_{c}}{A}\frac{2k_{m}k_{M}}{k_{m}+k_{M}}+\frac{A_{v}}{A}k_{g}\right]$. (S3)

After further simplifying this formula as a function of geometric position, it was compiled in C++.

In addition, the solute distribution within the cast-rolling zone was modeled using a solute transfer module, developed based on alloy composition and a comprehensive thermodynamic database in Table. S4, which was calculated through Thermo-Calc software. This module was intricately coupled with heat transfer, phase transformation, and flow conditions to provide a robust framework for capturing solute behavior under TRC's complex conditions. The core solute transport equations were derived from the Scheil solidification model, emphasizing the non-equilibrium solute segregation during rapid solidification:

$f_{l}\rho_{l}\frac{\partial c_{l}^{m}}{\partial t}+f_{l}\rho_{l}v_{l}\cdot\nabla c_{l}^{m}=\nabla\cdot\left(f_{l}\rho_{l}D_{l}^{m}\nabla c_{l}^{m}\right)+\left(c_{l}^{m}-c_{sl}^{m}\right)\frac{\partial}{\partial t}\left(\rho_{s}f_{s}\right)+\frac{S\rho_{s}D_{s}^{m}}{l}\left(c_{s}^{m}-c_{sl}^{m}\right)$, (S4)

where *m* is the solute species; *l* = *f*_s_*d*_2_/6 the diffusion length; *d*_2_ the secondary dendrite arm space; *S* = 2/*d*_2_ the interfacial area concentration; and *sl* and *D* the solid/liquid interface and diffusivity, respectively. We employed user-defined functions (UDF) to precisely define solute-sensitive solid-liquid equilibrium lines, including the partition coefficient, solute diffusivity, and eutectic phase transitions, under non-equilibrium conditions. These UDF allowed for accurate modeling of solute redistribution and dynamic behavior at critical stages such as eutectic transformations.

At the eutectic point, the UDF utilized the lever rule to define mass, energy, and solute sources, effectively modeling the material exchange between solid and liquid phases. Additionally, the model accounted for interfacial drag forces during solid-liquid interface movement, capturing the interaction between interface dynamics and fluid viscosity.

This solute field model represents an advancement over traditional approaches by incorporating the complex phase equilibria and non-linear solute transport phenomena typically neglected in sub-rapid solidification scenarios. Consequently, it provides a more detailed and accurate understanding of solute redistribution dynamics in the TRC process.

Table. S4 Microscopic parameters of Al-based alloys

| Microscopic parameters | Dendritic nucleation1 | Dendritic nucleation2 | Dendritic nucleation3 | Eutectic nucleation1 | Eutectic nucleation2 | Eutectic growth kinetics | Pearlite growth kinetics pre~factor | Ferrite growth kinetics pre-factor | Pearlite nucleation pre~factor | Difference between graphite and cementite |
| --- | --- | --- | --- | --- | --- | --- | --- | --- | --- | --- |
| Value | 10.0 | 3.0 | 10.0 | 500.0 | 2.0 | 5.0×10^-6^ | 1.7×10^-2^ | 1 | 5000000 | 11 |

To ensure the accuracy of solute field calculations, the precise liquidus and solidus temperatures must be used. In conventional Al-Cu alloys, the eutectic point is around 540 °C, but introducing low-melting-point elements like Li can shift this point significantly. In this study, the differential scanning calorimetry (DSC) curve of TRC-processed 2060 Al-Li alloy was measured (Fig. S2a), showing a solidus temperature of 495 °C and a liquidus of 648 °C. This results in a solid-liquid temperature range of 153 °C, characteristic of a wide solidification range aluminum alloy. Additionally, the Al-Cu eutectic phase diagram was recalculated using Thermo-Calc software to incorporate the effect of Li, revealing a revised solidification line at 495 °C (Fig. S2b). These data were employed to precisely define solute field parameters and describe the solidification behavior of the alloy.


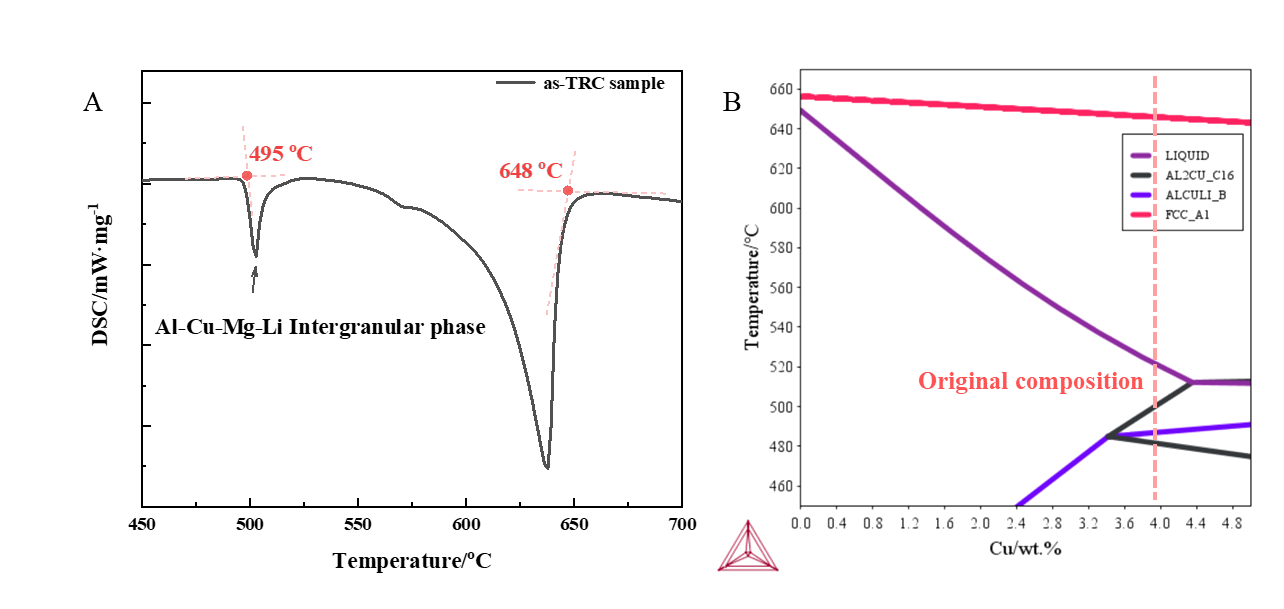


**Fig. S2 Determination of solidification parameters based on experiments and thermodynamic software.** Differential Scanning Calorimetry (DSC) analysis of TRC-processed 2060 Al-Li alloy (A). Revised Al-Cu-Li phase diagram calculated using Thermo-Calc software, accounting for the effect of Li addition, showing the solidus temperature at 495°C (B).

1. ***Simulation results***

Fig. S3 demonstrates the multi-field coupled simulation of the TRC process, highlighting the unique capability of our model to achieve full-process, realistic simulation, which is challenging due to the complexities involved in TRC's thermal-flow-solute interactions. The simulation captures multiple stages: early solidification (A), the onset of solidification (B), and the stable TRC stage (C), with each field—liquid fraction, thermal flow, and solute distribution—corresponding precisely at each stage. The solute field predictions effectively capture macrosegregation phenomena, showing that segregation originates from solute redistribution at the solid-liquid interface in the semi-solid region. Specifically, copper elements accumulate progressively at the interface. The accuracy of this solute field simulation lies in the implementation of composition-sensitive solid-liquid equilibrium lines compiled through UDF, which align well with the experimentally derived DSC results and ThermoCalc (V2023b)-calculated phase diagrams from Fig. S2. This alignment enabled accurate depiction of the eutectic behavior and composition partitioning based on lever rule principles, supporting the discussion on segregation behavior presented in the manuscript.

While eutectic behavior has also been implemented via UDF, the micro-scale redistribution effects are difficult to manifest clearly at the macro-scale in the solute field simulation, thus serving as support for model accuracy rather than a focus for extensive discussion.


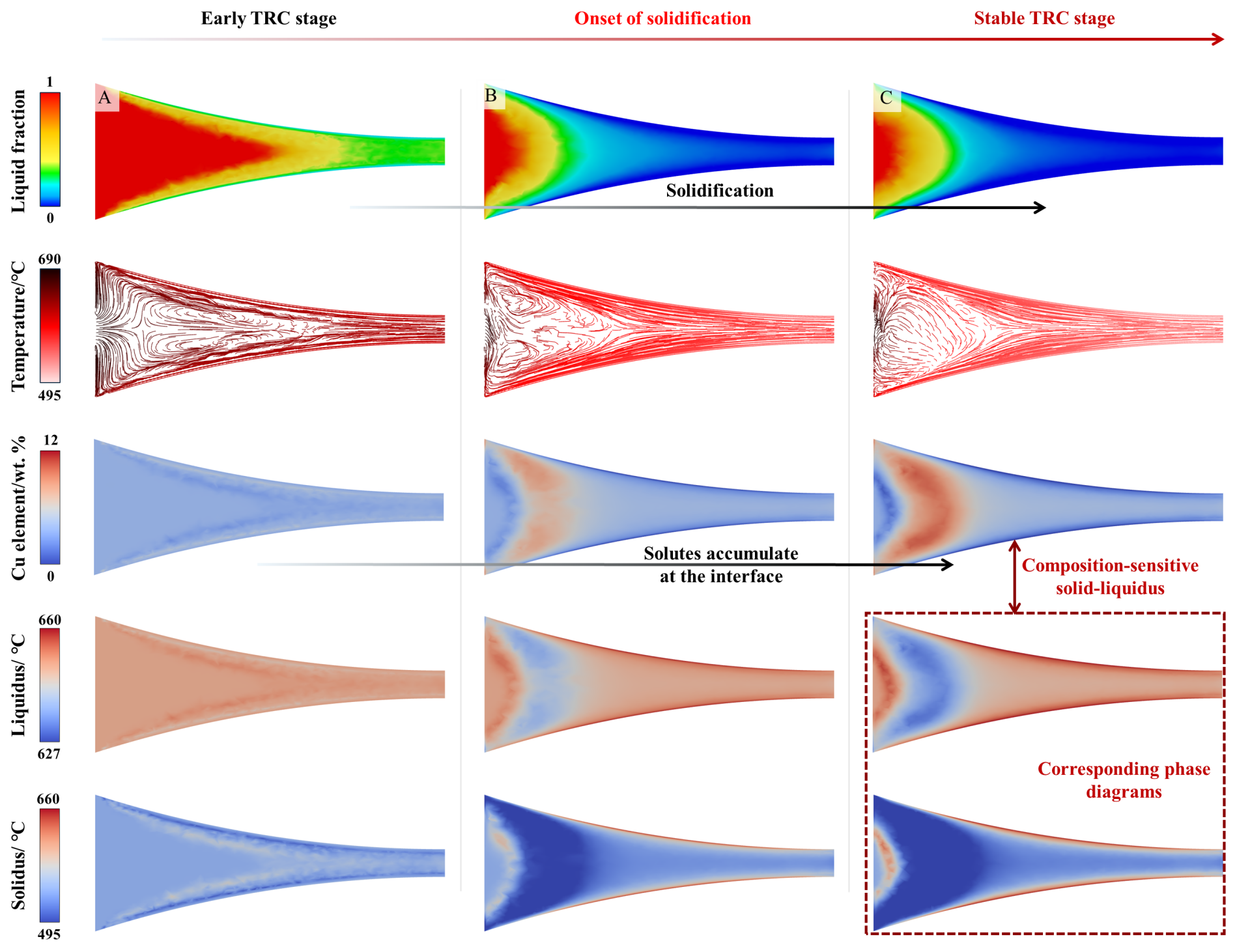


**Fig. S3 The multi-field coupled simulation of the TRC process.** The model developed through secondary programming, illustrates the evolution of heat, flow, and solute fields during various stages. The simulation captures the early solidification phase (A), onset of solidification (B), and the stable TRC stage (C), detailing liquid fraction, thermal flow, solute field distribution, and composition-sensitive behaviors corresponding to liquidus and solidus lines from the phase diagram.

1. ***Measurement of cooling speed***

To validate the sub-rapid solidification characteristics of the TRC process, a temperature measurement experiment was conducted. Prior to casting, K-type thermocouples, connected to a data logging system, were inserted into the casting-rolling zone (Fig. S4a). These thermocouples were rolled out along with the metal (Fig. S4b), enabling real-time monitoring of the temperature evolution during the casting-rolling process. By averaging multiple experimental curves, a cooling rate curve was obtained (Fig. S4c). The measured cooling rate between the liquidus and solidus temperatures was approximately 204.3 °C/s, indicating that the TRC process achieves a cooling rate characteristic of sub-rapid solidification. This experimental result provides direct validation of the sub-rapid solidification behaviors discussed in the manuscript, further demonstrating the potential of TRC in producing high-performance Al-Li alloys.


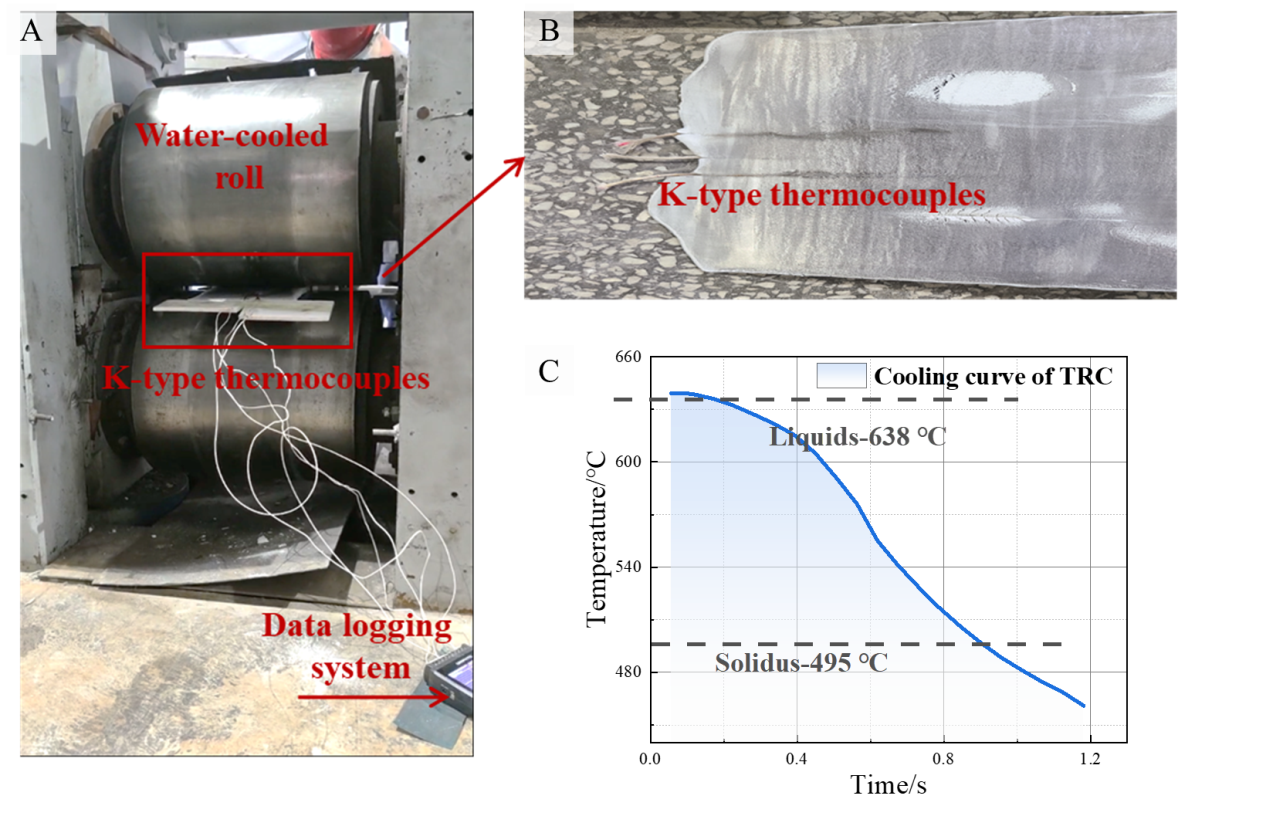


**Fig. S4 Validation of sub-rapid solidification characteristics in the TRC process.** Temperature measurement experiments were conducted by inserting K-type thermocouples connected to a data logging system into the cast-rolling zone prior to casting (A). The thermocouples were rolled out along with the metal strip during the TRC process (B). The cooling rate curve (C), averaged over multiple measurements, confirmed that the cooling rate reached a level characteristic of sub-rapid solidification, with values in the range of 204.3°C/s.

1. ***Microstructure before tensile test***

Fig. S5 serves as supplementary material to substantiate the microstructural analysis presented in the manuscript. Fig. S5a highlights the dispersed distribution of nanoclusters in the FA sample prior to dislocation interaction, supporting the observation that these clusters are uniformly distributed. Fig. S5b further emphasizes the complex atomic structure of the clusters, providing additional evidence of their enhanced solute tolerance, which is critical for the strengthening effect discussed in the manuscript.


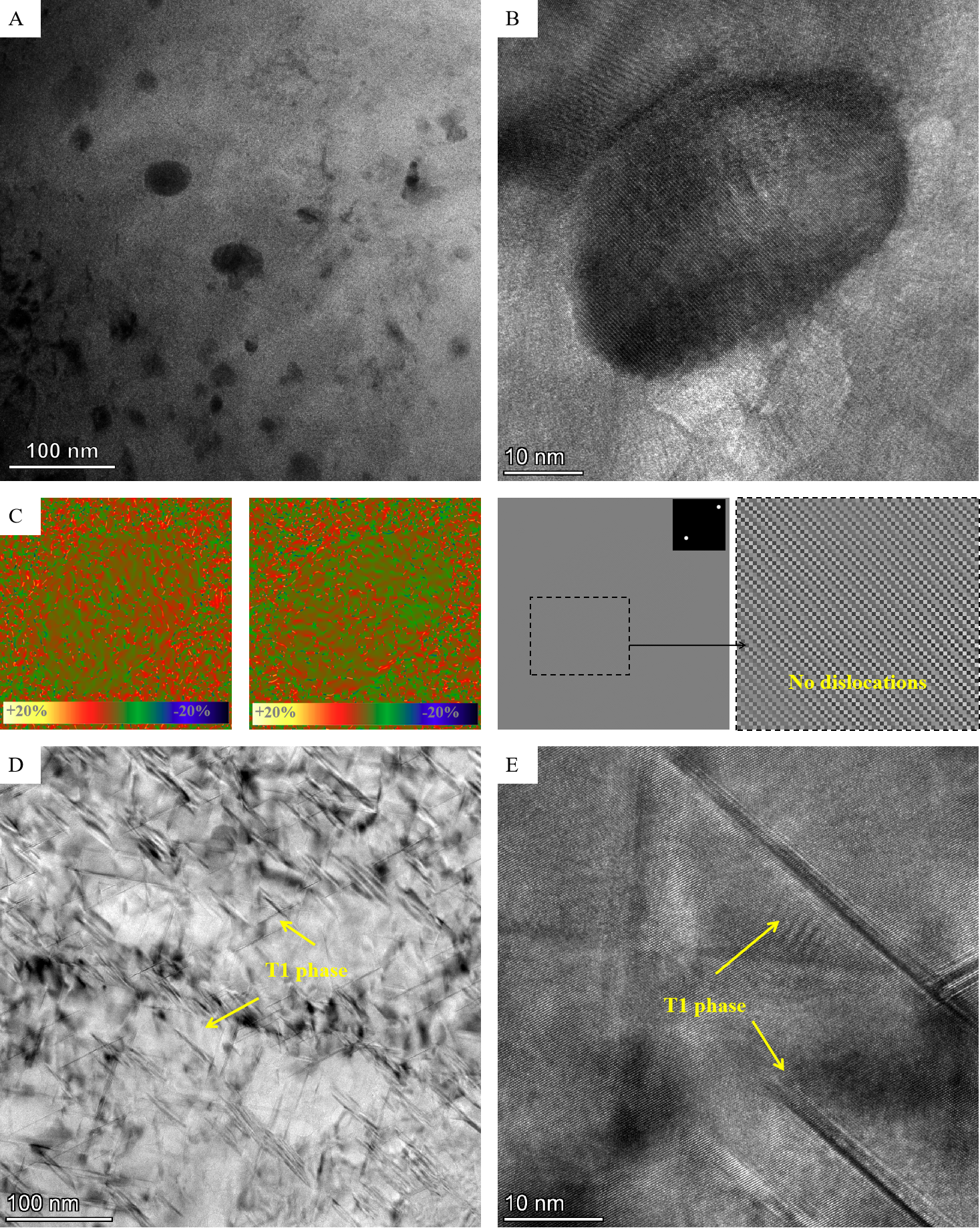


**Fig. S5 Microstructural characterization of the pre-tensile FA and SA samples.** The FA structure before tensile testing exhibits a large number of dispersed nanoclusters (A), with detailed atomic structures observed in magnified images (B), confirming the clusters' solute-tolerant nature. GPA analysis of strain distribution within the clusters and IFFT images are presented (C). The pre-tensile SA structure shows coexisting needle-like and coarsened phases (D), with magnified views revealing unsheared, heterogeneous needle and spherical phases (E).

The strain field analysis in Fig. S5c supports the assumptions made in Eq. 4 and 6 of the manuscript, where the stress influence area of the precipitates was considered approximately equal to their own size, as evidenced by the stress distribution in the strain maps. The inverse fast Fourier transform (IFFT) analysis in Fig. S5c also demonstrates the absence of partial atomic planes within the clusters when no dislocation passes through them, supporting the hypothesis that the partial atomic planes observed in Fig. 7 of the manuscript are associated with dislocations shearing through clusters.

Fig. S5d and e depict the heterogeneous distribution of needle-like phases coexisting with coarsened phases in the SA sample, even before dislocation passage. This phenomenon is attributed to the high segregation sensitivity of high-alloy compositions, further emphasizing the non-uniformity and instability of precipitate structures in the SA condition. These observations collectively provide strong evidence for the differences in microstructural evolution and subsequent strengthening mechanisms between FA and SA samples.

1. ***Microstructure after tensile test***

The fracture surface morphologies of FA and SA samples after tensile testing provide further insights into their respective mechanical behaviors. The FA sample exhibits predominantly ductile fracture characteristics, as shown by the presence of numerous dimples in Fig. S6a and b. These features indicate effective plastic deformation, consistent with the enhanced toughness and ductility previously discussed. In contrast, the SA sample displays a mixed fracture mode, characterized by both intergranular fracture and river patterns, as seen in Fig. S6c and d. This suggests localized stress concentration due to compositional segregation, which significantly contributed to reduced plasticity and brittle fracture behavior in SA.

The microstructural features observed in Fig. S7 provide supplementary evidence to support the findings presented in the main text regarding the distribution of precipitates in FA and SA samples after tensile deformation. Fig. S7a, b depict dislocations interacting with uniformly dispersed clusters in the FA sample, highlighting a more homogeneous microstructure that effectively mitigates stress concentration. In contrast, Fig. S7c, d illustrate dislocations interacting with the heterogeneous needle-like precipitates in the SA sample, showing irregular precipitate sizes and non-uniform distribution. This non-uniformity leads to localized stress concentration, contributing to a greater propensity for crack initiation and ultimately reducing the overall ductility of the material. These observations align with the mechanical behavior discussed in the main text, where the FA sample exhibited superior plasticity due to its more consistent microstructural arrangement.


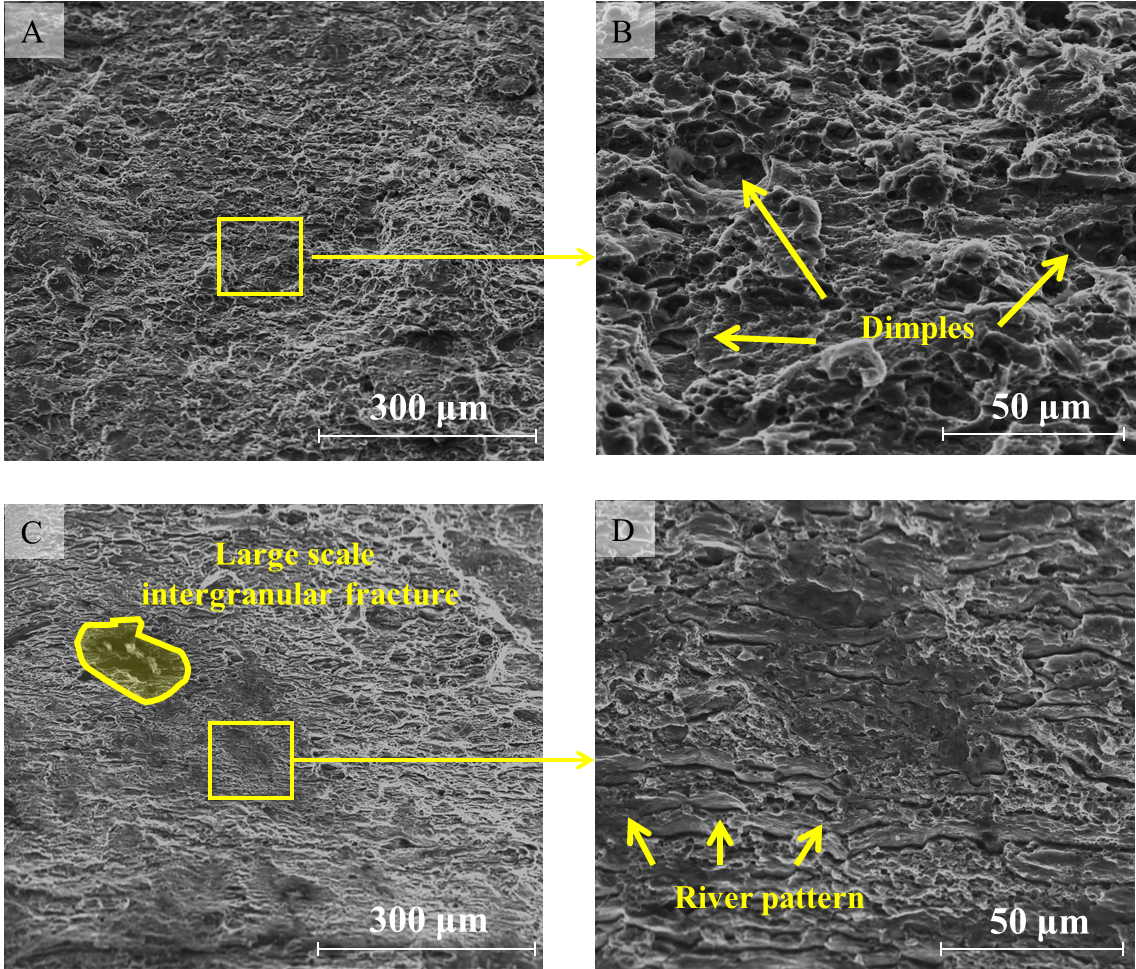


**Fig. S6 Microstructure of fracture surfaces after tensile testing.** The fracture morphologies of FA and SA samples are presented. (A) and (B) reveal typical ductile fracture characteristics in FA, with visible dimples. (C) and (D) illustrate the mixed fracture mode in SA, including intergranular fracture regions and river patterns, indicating brittle fracture behavior.


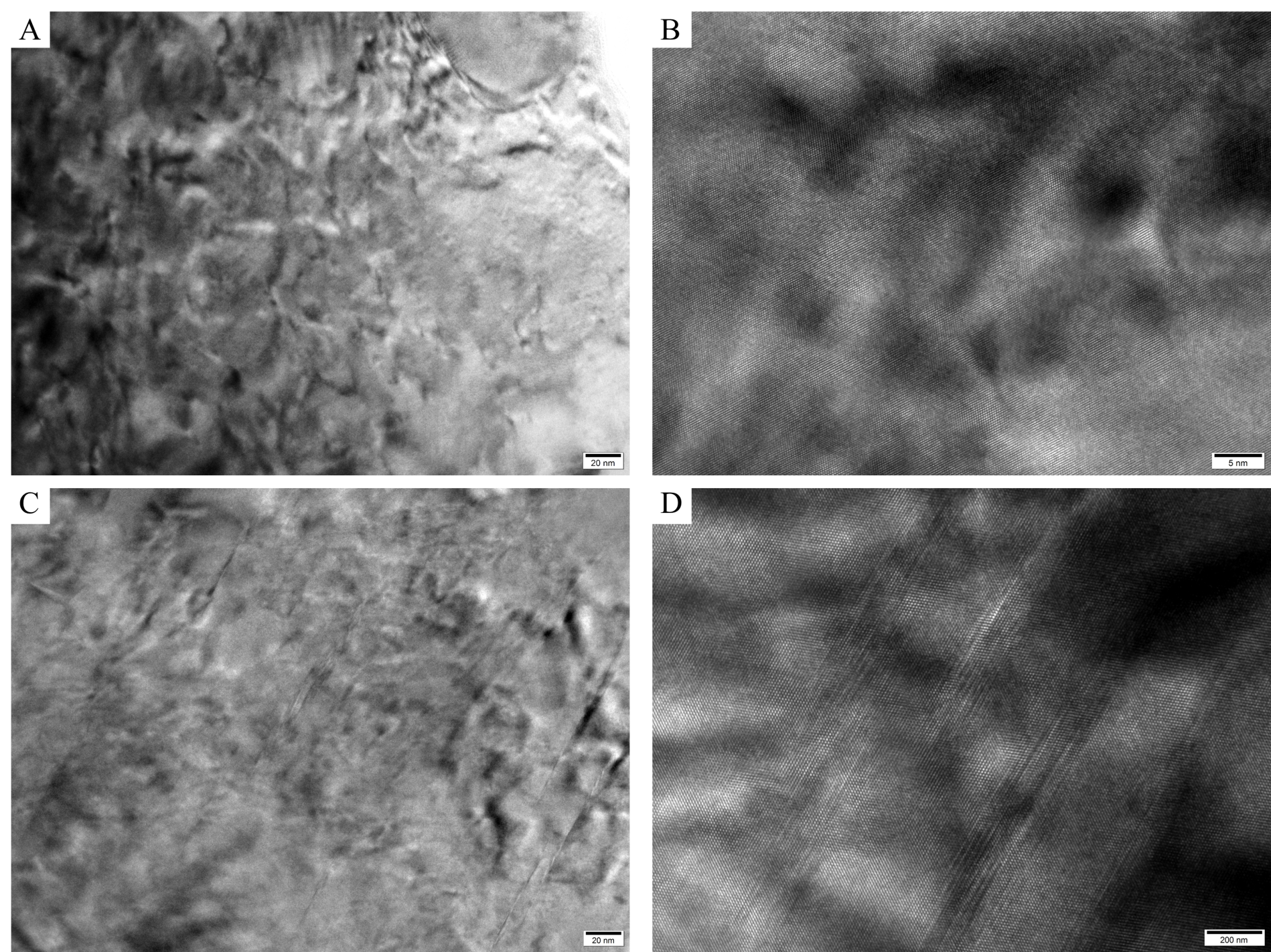


**Fig. S7 Post-tensile microstructural features at fracture locations.** Fig. S7 Post-tensile microstructural features at fracture locations. Dislocations interacting with dispersed clusters in the FA sample are observed in (A, B), while dislocations interacting with needle-like precipitates in the SA sample are depicted in (C, D).
